# High-frequency homogenization in periodic media with imperfect interfaces

**DOI:** 10.1098/rspa.2020.0402

**Published:** 2020-12-16

**Authors:** Raphaël C. Assier, Marie Touboul, Bruno Lombard, Cédric Bellis

**Affiliations:** 1 Department of Mathematics, The University of Manchester, Oxford Road, Manchester M13 9PL, UK; 2 Aix Marseille Univ, CNRS, Centrale Marseille, LMA, Marseille, France

**Keywords:** high-frequency homogenization, periodic media, imperfect interfaces

## Abstract

In this work, the concept of high-frequency homogenization is extended to the case of one-dimensional periodic media with imperfect interfaces of the spring-mass type. In other words, when considering the propagation of elastic waves in such media, displacement and stress discontinuities are allowed across the borders of the periodic cell. As is customary in high-frequency homogenization, the homogenization is carried out about the periodic and antiperiodic solutions corresponding to the edges of the Brillouin zone. Asymptotic approximations are provided for both the higher branches of the dispersion diagram (second-order) and the resulting wave field (leading-order). The special case of two branches of the dispersion diagram intersecting with a non-zero slope at an edge of the Brillouin zone (occurrence of a so-called Dirac point) is also considered in detail, resulting in an approximation of the dispersion diagram (first-order) and the wave field (zeroth-order) near these points. Finally, a *uniform approximation* valid for both Dirac and non-Dirac points is provided. Numerical comparisons are made with the exact solutions obtained by the Bloch–Floquet approach for the particular examples of monolayered and bilayered materials. In these two cases, convergence measurements are carried out to validate the approach, and we show that the uniform approximation remains a very good approximation even far from the edges of the Brillouin zone.

## 1. Introduction

Classically, dynamic homogenization is understood as a low-frequency approximation to wave propagation in heterogeneous media such as laminates, composites, or more generally any microstructured media. It consists in approximating such media by effective homogeneous media with specific properties. Homogenization is the mathematical process that allows one to find such properties. Much work on this topic has been carried out since the 1970s and it is not the aim of this introduction to be exhaustive in that regard. A particularly successful approach is the two-scale asymptotic expansion method and the notion of *slow* or *fast* variables (see e.g. [1–3]). Periodic media, of interest in this paper, are dealt with very efficiently by such a method.

In periodic media, waves can propagate at angular frequencies *ω* that are not necessarily small. The set of wavenumbers *k* (Bloch wavenumbers) at which waves propagate depends on the angular frequency through dispersion relations. In particular it can be shown that these dispersion relations can be entirely understood using diagrams restricting the Bloch wavenumbers to lie within the Brillouin zone. In one dimension, when the periodicity of the structure is *h* say, such a Brillouin zone is given by *k* ∈ [0, *π*/*h*]. Typically, the dispersion diagram displays band gaps, i.e. regions in the angular frequency space where waves cannot propagate. There tends to be infinitely many branches of the dispersion diagram, i.e. for a given Bloch wavenumber, one can find an infinite (countable) set of angular frequencies leading to propagating waves. Again, a lot has been written about this, and we do not aim to give an exhaustive literature review on this point, though we can refer the interested reader to [4] for example. See also [5] for a discussion of the band gaps in periodic materials from a physics point of view.

The idea of *high-frequency homogenization* is to approximate how the dispersion relation (and hence the media) will behave for angular frequencies *ω* that are close to the angular frequencies *ω*_0_ corresponding to an edge of the Brillouin zone on the dispersion diagram. In [6], a work that has largely inspired the present paper, Craster *et al*. applied a two-scale asymptotic expansion method in order to achieve this for perfect interfaces. Adopting the terminology of [7], we will refer to the homogenization near the left edge of the Brillouin zone *k* ≈ 0 as finite frequency low wavenumber (FFLW), while the homogenization near the right edge (*k* ≈ *π*/*h*) will be referred to as finite frequency finite wavenumber (FFFW). Upon introducing $\tilde{k}$ as

1.1 $$(\text{FFLW}):\tilde{k}=k\;\text{and}\;(\text{FFFW}):\tilde{k}=\frac{\pi }{h}-k,$$

the end result of the high-frequency homogenization technique is an approximation of the type

1.2 $$\omega^{2}=\omega_{0}^{2}+T{\left(\tilde{k}h\right)}^{2}+o\left(\frac{h^{2}{\tilde{k}}^{2}}{L^{2}}\right),$$

where *L* is a macroscopic characteristic length of the material and the parameter T∈R can be determined explicitly. This angular frequency approximation comes together with an associated leading-order approximation to the wave field *U*_*h*_ (*X*) of the form

1.3 $$U_{h}(X)=U_{h}^{\left(0\right)}(X)+O\left(\frac{h\tilde{k}}{L}\right).$$

One should note that in [7], the authors generalized the technique to work in any dimension, and also pushed the asymptotic work one order further than [6]. It is also worth mentioning the more recent work [8], in which the technique was developed while including a source term in the initial evolution equation. As shown in [9], high-frequency homogenization of wave equations in the time domain can also be carried out, and the methodology has also been applied in a discrete setting to structural mechanics [10] and elastic lattices [11].

In this work, following the approach of [6], we wish to focus on extending this high-frequency homogenization technique to one-dimensional periodic media that have an imperfect interface at the edges of the periodic cell, which, to our knowledge, has not been considered before.

Indeed, because of defects like air and cracks or thin layers of glue, for example, the contacts between solids are often not perfect, and a jump of the elastic stress and of the elastic displacement can occur across the contact area (the interface). There are different approaches when it comes to modelling such imperfect contact, and we refer to [12] for a comparison between different models. In particular, authors from various disciplines (e.g. non-destructive evaluation of materials or geophysics) have modelled such situations using the so-called *spring-mass* conditions that we will use in the present work. These conditions, satisfied by wave fields across an interface, are analogous to the mechanical laws of springs [13–16] or springs and masses [17,18]. Stiffness and mass values are expected to be connected, although not necessarily in a trivial way, to the contact quality [19,20]. More recently, rational derivations based on asymptotic expansion have been proposed, yielding similar spring-mass models of imperfect interface [21]. These imperfect conditions arise from a homogenization process of a thin interphase. The results presented in the paper are hence relevant for wavelength comparable with the periodicity, but much larger than the thin interphase approximated by the imperfect contact laws. Here, we restrict ourselves to linear imperfect contact, though we kindly refer the reader to our work [22] on low-frequency homogenization in the time-domain, where both linear and nonlinear imperfect contacts are considered. There exists a significant amount of work considering the homogenization of such materials in the static regime (see for example [23,24] and the more recent work [25]) but it seems that dynamic homogenization has seldom been treated.

The rest of the paper is organized as follows. In §2a we formulate the one-dimensional physical problem at hand, namely that of linear elastic wave propagation through a layered material with periodic, possibly space dependent, material properties. The non-dimensionalization of the problem is performed in §2b, leading to the introduction of our small parameter *δ*, which is fully exploited via the two-scale asymptotic expansion method in §2c. Sections 3a, 3b and 3c are respectively dedicated to the asymptotic expansion at order *δ*^0^, *δ*^1^ and *δ*^2^, before combining these results in §3d to explicitly obtain the zeroth-order approximated wave-field and the associated formula for the parameter T introduced in (1.2). Effectively, §3 provides approximations of both the dispersion diagram (second-order) and the wave-field (zeroth-order) at the edges of the band gaps. In the limiting case of a point on the edge of the Brillouin zone where two branches of the dispersion diagrams intersect with non-zero slope (such intersections are also known in the literature (see e.g. [7,26,27]) as *Dirac points* or *Dirac cones*), the method developed in §3 needs to be adapted. This is what is done in §4, resulting in a linear approximation to the dispersion diagram. In order to provide a smooth transition between the asymptotics of §§3 and 4, we consider the intermediate case of narrow band gaps in §5 and obtain a *uniform approximation* that remains valid in the Dirac point limit.

We then illustrate the method on two concrete examples, a homogeneous material (§6a) and a bilayered material (§6b) with periodically distributed imperfect interfaces. Such examples are also treated by the Bloch–Floquet analysis (see appendix B), which allows us to illustrate and quantify the validity of our method. In particular, we discuss the occurrence of Dirac points, for which the approach of §4 should be taken. It is shown that the asymptotic expansion of §3 is a good local approximation near the edges of the band gaps, and that, even for not so narrow band gaps, the uniform approximation of §5 performs extremely well for almost the entirety of a given branch of the dispersion diagram. Perspectives and conclusions are given in §7.

## 2. Problem formulation

### (a) The physical problem

**Figure 1.**
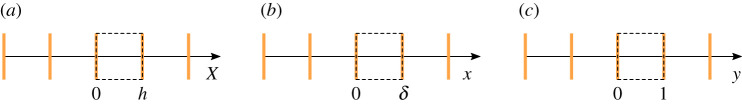
Geometry settings in the (*a*) *X*, (*b*) *x* and (*c*) *y* variables, a periodic cell is highlighted with a dashed line. (Online version in colour.)

We consider linear elastic waves propagation at a given angular frequency *ω* through a periodic medium of periodicity *h* > 0 and with a macroscopic characteristic length *L* > 0. We denote the physical space variable *X*; the density *ρ*_*h*_ (*X*) and the Young’s modulus *E*_*h*_ (*X*) of the elastic medium are assumed to be *h*-periodic piecewise smooth, *L*^∞^ and strictly positive. We may assume without loss of generalities that the edges of the periodic cell are located at *X*_*n*_ = *nh* for n∈Z, as illustrated in figure 1*a*. We further assume that the interfaces across the edges of the periodic cells are imperfect, and of the linear spring-mass type, characterized by some mass and stiffness parameters denoted respectively *M* and *K* that are both strictly positive. This results in the following governing equation and jump conditions for the displacement field *U*_*h*_ (*X*):

2.1 $$\frac{\text{d}}{\text{d}X}\left(E_{h}(X)\frac{\text{d}U_{h}}{\text{d}X}\right)+\rho_{h}(X)\omega^{2}U_{h}=0,\;\text{with }\;\left\{ \begin{array}{l} \text{⟦}U_{h}{\text{⟧}}_{X_{n}}=\frac{1}{K}{\left\langle E_{h}\frac{\text{d}U_{h}}{\text{d}X}\right\rangle }_{X_{n}},\\ {\left[\left[E_{h}\frac{\text{d}U_{h}}{\text{d}X}\right]\right]}_{X_{n}}=-M\omega^{2}\langle \langle U_{h}{\rangle \rangle }_{X_{n}}, \end{array}\right.$$

where $\text{⟦}\cdot {\text{⟧}}_{X_{n}}$ and ${\left\langle \langle \cdot \rangle \right\rangle }_{X_{n}}$ are respectively called the jump and mean brackets at the interface *X*_*n*_, and are defined for any function *g* (*X*) by

2.2 $$\text{⟦}g{\text{⟧}}_{X_{n}}=g(X_{n}^{+})-g(X_{n}^{-})\;\text{and}\;{\left\langle \langle g\rangle \right\rangle }_{X_{n}}=\frac{1}{2}(g(X_{n}^{+})+g(X_{n}^{-})).$$

Due to the *h*-periodicity of *ρ*_*h*_ and *E*_*h*_, it is possible to write them as *ρ*_*h*_ (*X*) = *ρ*(*X*/*h*) and *E*_*h*_ (*X*) = *E* (*X*/*h*) for some 1-periodic functions *ρ* and *E*.

**Note 2.1.**

Throughout this work, we assume that any discontinuity of *ρ* and *E* on (0, 1) are of a perfect contact nature. This means that apart from at the interfaces *X* = *X*_*n*_, we will assume that the displacement *U*_*h*_ (*X*) and the stress *E*_*h*_ (*X*) (d*U*_*h*_/d*X*) are continuous.

Classically, such wave propagation problem in periodic media can be understood by using the so-called Bloch–Floquet analysis that consists in seeking solutions of the form

$$U_{h}(X)=U_{h}(X)\;{\text{e}}^{\text{i}kX},$$

that propagate at the Bloch wavenumber *k*, and where $U_{h}(X)$ is *h*-periodic, that is, $U_{h}(X+h)=U_{h}(X)$. In certain simple cases (see e.g. §6a,b), this leads to an explicit dispersion relation relating *ω* to *k*, the graphical representation of which is the so-called dispersion diagram.

One should note that for *k* = 0, we have *U*_*h*_ (*X*) = *U*_*h*_ (*X* + *h*), i.e. the solution is periodic, while for *k* = *π*/*h*, we have *U*_*h*_ (*X*) = −*U*_*h*_ (*X* + *h*), i.e. the solution is antiperiodic. Such values of *k* correspond to the edges of the so-called Brillouin zone. The aim of the present work is to approximate the Bloch–Floquet solutions propagating at wavenumbers that are close to *k* = 0 (FFLW) or $k=\frac{\pi }{h}$ (FFFW) with finite angular frequency *ω* = *O* (1).

### (b) Non-dimensionalization

In order to simplify the mathematical notations, we start by non-dimensionalizing the physical problem (2.1). In order to do so, we define the characteristic dimensional density $\rho^{⋆}=\langle \rho \rangle$, Young’s modulus $E^{⋆}={\left\langle 1/E\right\rangle }^{-1}$ and wavespeed $c^{⋆}=\sqrt{E^{⋆}/\rho^{⋆}}$, where the average operator 〈 · 〉 is defined for any function *g* as

2.3 $$\langle g\rangle =\int_{0}^{1}g(y)\;\text{d}y.$$

These can be used in order to define the following non-dimensional quantities

2.4 $$x=\frac{X}{L},\;\delta =\frac{h}{L},\;\mu =\frac{\omega h}{c^{⋆}},\;\kappa =Lk,\;\alpha =\frac{\rho }{\rho^{⋆}},\;\beta =\frac{E}{E^{⋆}},\;k=\frac{Kh}{E^{⋆}},\;m=\frac{M}{h\rho^{⋆}},\;u_{\delta }(x)=\frac{U_{h}(X)}{L}.$$

The starred quantities $\rho^{⋆}$ and $E^{⋆}$ are chosen for convenience to be the effective properties of the medium obtained by low-frequency homogenization, implying that 〈*α*〉 = 〈1/*β*〉 = 1, but this choice is somewhat arbitrary. Moreover, we can also show that

$$\rho_{h}(X)=\rho \left(\frac{X}{h}\right)=\rho \left(\frac{x}{\delta }\right)\;\text{and}\;E_{h}(X)=E\left(\frac{X}{h}\right)=E\left(\frac{x}{\delta }\right).$$

Using these quantities, (2.1) can be rewritten as the non-dimensional governing equation

2.5 $$\delta^{2}\frac{\text{d}}{\text{d}x}\left(\beta \left(\frac{x}{\delta }\right)\frac{\text{d}u_{\delta }}{\text{d}x}(x)\right)+\mu^{2}\alpha \left(\frac{x}{\delta }\right)u_{\delta }(x)=0,$$

subject to the jump conditions

2.6 $$\text{⟦}u_{\delta }{\text{⟧}}_{x_{n}}=\frac{\delta }{k}{\left\langle \left\langle \beta \left(\frac{x}{\delta }\right)\frac{\text{d}u_{\delta }}{\text{d}x}\right\rangle \right\rangle }_{x_{n}}\;\text{and}\;\delta {〚\beta \left(\frac{x}{\delta }\right)\frac{\text{d}u_{\delta }}{\text{d}x}〛}_{x_{n}}=-m\mu^{2}{\left\langle \langle u_{\delta }\rangle \right\rangle }_{x_{n}},$$

in the geometry setting of figure 1*b*. The equations (2.5)–(2.6) constitute our non-dimensional problem. Note that in this non-dimensional setting, the Bloch–Floquet analysis is still valid, and consists in looking for solutions of the form

2.7 $$u_{\delta }(x)=u_{\delta }(x)\;{\text{e}}^{\text{i}\kappa x},$$

where *κ* is the non-dimensional Bloch wavenumber and $u_{\delta }$ is *δ*-periodic. Note that this implies that *u*_*δ*_ and its derivative *u*_*δ*_′ satisfy

2.8 $$u_{\delta }(x+\delta )=u_{\delta }(x)\;{\text{e}}^{\text{i}\kappa \delta }\;\text{and}\;u_{\delta }^{\prime }(x+\delta )=u_{\delta }^{\prime }(x)\;{\text{e}}^{\text{i}\kappa \delta }.$$

**Remark 2.2.**

The FFLW case corresponds to *κδ* ≈ 0 and the FFFW case corresponds to *κδ* ≈ *π*. When *κδ* is exactly 0 (resp. *π*), then the solution *u*_*δ*_ (*x*) is *δ*-periodic (resp. *δ*-antiperiodic).

### (c) Two-scale asymptotic expansion

We will now make the assumption that the macroscopic characteristic length *L* is much bigger than the periodicity *h*, implying that *δ* ≪ 1. The material parameters *α* and *β* will hence vary on a fine scale associated with the rescaled coordinate *y* = *x*/*δ* (see figure 1*c* for the associated geometrical configuration).

Following the two-scale expansion technique, we further assume that the displacement field will have small scale features described by *y*, and slow continuous variations described by *x*. We hence pose the following ansatz for the wave field *u*_*δ*_ and the reduced frequency *μ*:

2.9 $$u_{\delta }(x)=\sum_{j\geq 0}\delta^{j}u_{j}(x,y)\;\text{and}\;\mu^{2}=\sum_{\ell \geq 0}\delta^{\ell }\mu_{\ell }^{2},$$

and treat *x* and *y* as two independent variables (scale separation), implying that d/d*x* ↔ ∂/∂*x* + (1/*δ*) (∂/∂*y*). In the FFLW case we will assume that *u*_*j*_ is 1-periodic in *y*, that is *u*_*j*_ (*x*, *y*) = *u*_*j*_ (*x*, *y* + 1), while in the FFFW case, we will assume that *u*_*j*_ is 1-antiperiodic, that is *u*_*j*_ (*x*, *y*) = −*u*_*j*_ (*x*, *y* + 1), see remark 2.2. In what follows, we will treat both cases simultaneously.

The non-dimensional problem (2.5)–(2.6) can hence be rewritten as the governing equation

2.10 $$\sum_{j\geq 0}\left[\delta^{j}\frac{\partial }{\partial y}\left(\beta \frac{\partial u_{j}}{\partial y}\right)+\delta^{j+1}\left\{\beta \frac{\partial^{2}u_{j}}{\partial x\partial y}+\frac{\partial }{\partial y}\left(\beta \frac{\partial u_{j}}{\partial x}\right)\right\}+\delta^{j+2}\beta \frac{\partial^{2}u_{j}}{\partial x^{2}}+\sum_{\ell \geq 0}\delta^{\ell +j}\mu_{\ell }^{2}\alpha u_{j}\right]=0,$$

subject to the jump conditions at *y*_*n*_ = *n*:

2.11 $$\sum_{j\geq 0}\delta^{j}\text{⟦}u_{j}(x,y){\text{⟧}}_{y_{n}}=\frac{\delta }{k}\sum_{j\geq 0}\delta^{j}{\left\langle \left\langle \beta (y)\left(\frac{\partial u_{j}}{\partial x}+\frac{1}{\delta }\frac{\partial u_{j}}{\partial y}\right)\right\rangle \right\rangle }_{y_{n}}$$

and

2.12 $$\delta \sum_{j\geq 0}\delta^{j}{〚\beta (y)\left(\frac{\partial u_{j}}{\partial x}+\frac{1}{\delta }\frac{\partial u_{j}}{\partial y}\right)〛}_{y_{n}}=-m\left(\sum_{\ell \geq 0}\delta^{\ell }\mu_{\ell }^{2}\right)\left(\sum_{j\geq 0}\delta^{j}{\left\langle \langle u_{j}\rangle \right\rangle }_{y_{n}}\right),$$

where for any function *g* (*x*, *y*), the jump and mean brackets are naturally defined for n∈Z as

2.13 $$\text{⟦}g(x,y){\text{⟧}}_{y_{n}}=g(x,n^{+})-g(x,n^{-})\;\text{and}\;{\left\langle \langle g\rangle \right\rangle }_{y_{n}}=\frac{1}{2}(g(x,n^{+})+g(x,n^{-})).$$

**Note 2.3.**

In order for our expansion to be compatible with the assumption regarding potential discontinuities of *ρ* and *E* within the unit cell made in note 2.1, we seek the fields such that *u*_0_, *β*(∂*u*_0_/∂*y*), *u*_*j*_ and *β*(∂*u*_*j*_/∂*y* + ∂*u*_*j*−1_/∂*x*) for *j* ≥ 1 are continuous functions of *y* on (0, 1).

Before pushing the asymptotic analysis further, we need to discuss some important properties of the jump and mean brackets. We will start with a very useful property (that can be proved directly), namely that for any two functions *f* (*x*, *y*) and *g* (*x*, *y*), and any n∈Z, the following relation is valid:

2.14 $$\text{⟦}fg{\text{⟧}}_{y_{n}}=\text{⟦}f{\text{⟧}}_{y_{n}}{\left\langle \langle g\rangle \right\rangle }_{y_{n}}+{\left\langle \langle f\rangle \right\rangle }_{y_{n}}\text{⟦}g{\text{⟧}}_{y_{n}}.$$

It is equally important to note that if the function subjected to the brackets is either periodic or antiperiodic, the following result holds.

**Lemma 2.4.**

*Let* n∈Z. *If a function* $g^{\mathrm{per}}(x,y)$ *is 1-periodic in* *y*, *then we can write*

$$\text{⟦}g^{\mathrm{per}}{\text{⟧}}_{y_{n}}=g^{\mathrm{per}}(x,0^{+})-g^{\mathrm{per}}(x,1^{-})\;\text{and}\;{\left\langle \langle g^{\mathrm{per}}\rangle \right\rangle }_{y_{n}}=\frac{1}{2}(g^{\mathrm{per}}(x,0^{+})+g^{\mathrm{per}}(x,1^{-})),$$

*while for a function* $g^{\mathrm{anti}}(x,y)$ *that is 1-antiperiodic in* *y*, *we have*

$$\text{⟦}g^{\mathrm{anti}}{\text{⟧}}_{y_{n}}=g^{\mathrm{anti}}(x,0^{+})+g^{\mathrm{anti}}(x,1^{-})\;\text{and}\;{\left\langle \langle g^{\mathrm{anti}}\rangle \right\rangle }_{y_{n}}=\frac{1}{2}(g^{\mathrm{anti}}(x,0^{+})-g^{\mathrm{anti}}(x,1^{-})).$$

The lemma 2.4 means that as long as the function subjected to either the jump or the mean bracket is 1-periodic or 1-antiperiodic in *y*, then the mean and jump brackets values are independent of *n*, and the *y*_*n*_ subscript can be dropped. Since *β*(*y*) is 1-periodic and *u*_*j*_(*x*, *y*) is either 1-periodic or 1-antiperiodic in *y*, this is the case for all the brackets in the conditions (2.11) and (2.12), we will hence drop the subscript from now on and just use ⟦⋅⟧ and ⟨⟨⋅⟩⟩. We will make sure that whenever this notation is used, the function inside the brackets is either periodic or antiperiodic. In particular, from lemma 2.4 and (2.14), we obtain directly the following lemma that will prove very important in what follows.

**Lemma 2.5.**

*For any two functions* *f* (*x*, *y*) *and* *g* (*x*, *y*) *that are either 1-periodic or 1-antiperiodic in y, we have* ⟦fg⟧=⟦f⟧⟨⟨g⟩⟩+⟨⟨f⟩⟩⟦g⟧.

Finally, in order to link the average operator and the jump bracket, the following result will be very useful.

**Lemma 2.6.**

*For any function* $g^{\mathrm{per}}(x,y)$ *that is 1-periodic and continuous for* *y* ∈ (0, 1), *we have*

$$\left\langle \frac{\partial g^{\mathrm{per}}}{\partial y}\right\rangle =-\text{⟦}g^{\mathrm{per}}\text{⟧}.$$

**Notation 2.7.**

From now on, in order to efficiently deal with the FFLW (*κδ* ≈ 0) and the FFFW (*κδ* ≈ *π*) cases simultaneously, we will assume that whenever the symbols ± or ∓ are used, the top sign corresponds to FFLW while the bottom sign corresponds to FFFW.

We are now well equipped to start developing the core theoretical part of the paper.

## 3. The case of simple eigenvalues

We will now deploy the two-scale asymptotic procedure in order to derive the approximation (1.2). In order to do so we will need to consider the contributions of (2.10)–(2.12) at the orders *δ*^0^, *δ*^1^ and *δ*^2^.

### (a) Zeroth-order field

Upon collecting the terms of order *δ*^0^ in (2.10)–(2.12), we obtain the system

3.1 $$(3.1a)\;\frac{\partial }{\partial y}\left(\beta \frac{\partial u_{0}}{\partial y}\right)+\mu_{0}^{2}\alpha u_{0}=0\;\text{with}\;(3.1b)\;\left\{ \begin{array}{l} \text{⟦}u_{0}\text{⟧}=\frac{1}{k}\langle \langle \beta \frac{\partial u_{0}}{\partial y}\rangle \rangle ,\\ {}[[\beta \frac{\partial u_{0}}{\partial y}]]=-m\mu_{0}^{2}\langle \langle u_{0}\rangle \rangle . \end{array}\right.$$

Upon considering the (Sturm–Liouville) differential operator L:=(−1/α)(d/dy)(β(d/dy)), one can see that the system (3.1) constitutes an eigenvalue problem for L. It is a bit unusual since the eigenvalue also appears in the boundary conditions, but one can show, using the tailored inner product 〈 · , · 〉 defined for some functions *f*(*y*) and *g*(*y*) (either both FFLW or both FFFW) by

3.2 $$\langle \;f,g\rangle =\langle \alpha f\bar{g}\rangle +m\langle \langle f\rangle \rangle \langle \langle \bar{g}\rangle \rangle ,$$

that this operator is symmetric (i.e. self-adjoint) and non-negative in both the FFLW and FFFW cases, the proof being deferred to appendix A. Therefore, it has a discrete set of (possibly repeated) real positive eigenvalues associated with real eigenfunctions. We denote the square root of such eigenvalues by *μ*_0_, and we note that eigenfunctions associated to different FFLW (resp. FFFW) eigenvalues are orthogonal for the inner product (3.2). These reduced frequencies *μ*_0_ correspond to the intersection of the dispersion diagram with the left (FFLW) or the right (FFFW) border of the Brillouin zone.

From now on we will choose one of these eigenvalues, denote it by *μ*_0_, and endeavour to approximate the solutions for some parameters $\left(\mu ,\tilde{\kappa }\right)$ close to (*μ*_0_, 0), where we define $\tilde{\kappa }$ to be

3.3 $$(\text{FFLW}):\tilde{\kappa }=\kappa \;\text{and}\;(\text{FFFW}):\tilde{\kappa }=\frac{\pi }{\delta }-\kappa ,$$

allowing us to treat the FFLW and FFFW cases simultaneously. We will also assume that *μ*_0_ is a simple eigenvalue (multiplicity 1). The case of a double eigenvalue will be dealt with in §4. Hence, there is only one eigenfunction that we denote ${\hat{u}}_{0}(y)$ and that is either periodic (FFLW) or antiperiodic (FFFW). The associated solution *u*_0_ to (3.1) can therefore be rewritten

3.4 $$u_{0}(x,y)=U_{0}(x){\hat{u}}_{0}(y),$$

for some function $U_{0}(x)$. It is worth mentioning at this stage that when inputting (3.4) into (3.1), we find that ${\hat{u}}_{0}(y)$ satisfies both the equation (3.1*a*) and the jump conditions (3.1*b*), a fact that will be used throughout the paper.

The main aim of what follows is to derive a differential equation with constant coefficients satisfied by $U_{0}(x)$. Note that in the case of low-frequency homogenization, the zeroth-order field *u*_0_ (*x*, *y*) can be shown to be independent of *y*; this is one of the main differences between low- and high-frequency homogenization.

### (b) First-order field

Let us now set *μ*_0_ to be one of the reduced frequencies found in the previous section, we can collect the terms of order *δ*^1^ in (2.10)–(2.12) to obtain the following system governing the first-order field *u*_1_:

3.5 $$\frac{\partial }{\partial y}\left(\beta \left(\frac{\partial u_{1}}{\partial y}+\frac{\partial u_{0}}{\partial x}\right)\right)+\beta \frac{\partial^{2}u_{0}}{\partial x\partial y}+\alpha (\mu_{0}^{2}u_{1}+\mu_{1}^{2}u_{0})=0,$$

subject to the jump conditions

3.6 $$\text{⟦}u_{1}\text{⟧}=\frac{1}{k}\left\langle \left\langle \beta \left(\frac{\partial u_{0}}{\partial x}+\frac{\partial u_{1}}{\partial y}\right)\right\rangle \right\rangle \;\text{and}\;\left[\left[\beta \left(\frac{\partial u_{0}}{\partial x}+\frac{\partial u_{1}}{\partial y}\right)\right]\right]=-m(\mu_{0}^{2}\langle \langle u_{1}\rangle \rangle +\mu_{1}^{2}\langle \langle u_{0}\rangle \rangle ).$$

We will show below that there is only one possible value of *μ*_1_, and that it has to be zero.

#### (i) Proving that *μ*_1_ = 0

Following [6], we consider $\left\langle u_{1}\times (3.1\text{a})-u_{0}\times \text{(}3.5)\right\rangle$. The terms in $\alpha \mu_{0}^{2}u_{0}u_{1}$ cancel out, and using the fact that

$$u_{1}\frac{\partial }{\partial y}\left(\beta \frac{\partial u_{0}}{\partial y}\right)-u_{0}\frac{\partial }{\partial y}\left(\beta \left(\frac{\partial u_{1}}{\partial y}+\frac{\partial u_{0}}{\partial x}\right)\right)=\frac{\partial }{\partial y}\left(u_{1}\beta \frac{\partial u_{0}}{\partial y}-u_{0}\beta \left(\frac{\partial u_{1}}{\partial y}+\frac{\partial u_{0}}{\partial x}\right)\right)+\beta \frac{\partial u_{0}}{\partial y}\frac{\partial u_{0}}{\partial x},$$

we obtain

3.7 $$\left\langle \frac{\partial }{\partial y}\left(u_{1}\beta \frac{\partial u_{0}}{\partial y}-u_{0}\beta \left(\frac{\partial u_{1}}{\partial y}+\frac{\partial u_{0}}{\partial x}\right)\right)\right\rangle =\underset{=0}{\underbrace{\left\langle \beta \left(u_{0}\frac{\partial^{2}u_{0}}{\partial x\partial y}-\frac{\partial u_{0}}{\partial x}\frac{\partial u_{0}}{\partial y}\right)\right\rangle }}+\mu_{1}^{2}\langle \alpha u_{0}^{2}\rangle .$$

Note that due to the form (3.4) of *u*_0_, we have *u*_0_ (∂^2^ *u*_0_/∂*x*∂*y*) − (∂*u*_0_/∂*x*) (∂*u*_0_/∂*y*) = 0, so the first bracket in the right-hand side (RHS) of the equation above is actually zero. Now, using note 2.3 and lemma 2.6, (3.7) becomes

3.8 $$-\left[\left[u_{1}\beta \frac{\partial u_{0}}{\partial y}-u_{0}\beta \left(\frac{\partial u_{1}}{\partial y}+\frac{\partial u_{0}}{\partial x}\right)\right]\right]=\mu_{1}^{2}\langle \alpha u_{0}^{2}\rangle .$$

When perfect interfaces are considered, the jump bracket term in (3.8) is automatically zero, a fact that is used in [6] to conclude that *μ*_1_ = 0. Such reasoning cannot be used directly in our case. Instead, make use of lemma 2.5 to rewrite (3.8) as

3.9 $$\begin{array}{ll} \mu_{1}^{2}\langle \alpha u_{0}^{2}\rangle & =-[[u_{1}]]\langle \;\langle \beta \frac{\partial u_{0}}{\partial y}\rangle \;\rangle -\langle \;\langle u_{1}\rangle \;\rangle \left[\;\left[\beta \frac{\partial u_{0}}{\partial y}\right]\;\right]\\ & \;+[[u_{0}]]\langle \;\langle \beta \left(\frac{\partial u_{1}}{\partial y}+\frac{\partial u_{0}}{\partial x}\right)\rangle \;\rangle +\langle \;\langle u_{0}\rangle \;\rangle \left[\;\left[\beta \left(\frac{\partial u_{1}}{\partial y}+\frac{\partial u_{0}}{\partial x}\right)\right]\;\right]\\ & =-k[[u_{0}]][[u_{1}]]+m\mu_{0}^{2}\langle \;\langle u_{1}\rangle \;\rangle \langle \;\langle u_{0}\rangle \;\rangle +k[[u_{0}]][[u_{1}]]-m\langle \;\langle u_{0}\rangle \;\rangle (\mu_{0}^{2}\langle \;\langle u_{1}\rangle \;\rangle +\mu_{1}^{2}\langle \;\langle u_{0}\rangle \;\rangle ), \end{array}$$

where the jump conditions (3.1*a*) and (3.6) have been used. Many terms cancel out and we get

3.10 $$\mu_{1}^{2}(\langle \alpha u_{0}^{2}\rangle +m\langle \langle u_{0}{\rangle \rangle }^{2})=0,\;\text{implying that}\;\mu_{1}=0,$$

because m and *α* are strictly positive, and, in the representation (3.4), $U_{0}$ cannot be identically zero and ${\hat{u}}_{0}$ is a real function. This result is very important and lies at the heart of the success of the high-frequency homogenization method.

#### (ii) An expression for *u*_1_ (*x*, *y*)

We can now simplify the equation (3.5) governing *u*_1_ to

3.11 $$\frac{\partial }{\partial y}\left(\beta \frac{\partial u_{1}}{\partial y}\right)+\alpha \mu_{0}^{2}u_{1}=-U_{0}^{\prime }(x)(2\beta (y){\hat{u}}_{0}^{\prime }(y)+\beta^{\prime }(y){\hat{u}}_{0}(y)).$$

Upon noting that, because ${\hat{u}}_{0}$ is a solution to (3.1*a*), the field $-yU_{0}^{\prime }(x){\hat{u}}_{0}(y)$ is a particular solution to (3.11), and that the differential operator applied to *u*_1_ is exactly the same as that of (3.1*a*), we can conclude that *u*_1_ can be written as

3.12 $$u_{1}(x,y)=U_{1}(x){\hat{u}}_{0}(y)+U_{0}^{\prime }(x)(v_{1}(y)-y{\hat{u}}_{0}(y)),$$

for some function $U_{1}(x)$ that will be shown not to play any role in what follows, and a function $v_{1}(y)$, which is another solution to (3.1*a*), independent of ${\hat{u}}_{0}(y)$, and that is chosen to ensure that the jump conditions

3.13 $$\text{⟦}u_{1}\text{⟧}=\frac{1}{k}\left\langle \left\langle \beta \left(\frac{\partial u_{0}}{\partial x}+\frac{\partial u_{1}}{\partial y}\right)\right\rangle \right\rangle \;\text{and}\;\left[\left[\beta \left(\frac{\partial u_{0}}{\partial x}+\frac{\partial u_{1}}{\partial y}\right)\right]\right]=-m\mu_{0}^{2}\langle \langle u_{1}\rangle \rangle$$

are satisfied. These jump conditions come from (3.6), where we have used that *μ*_1_ = 0. Note that because of (3.12), and the periodicity properties of *u*_1_ and ${\hat{u}}_{0}$, the function $v_{1}(y)-y{\hat{u}}_{0}(y)$ has to be periodic (FFLW) or antiperiodic (FFFW). Because this function will appear many times in what follows, it is worth giving it a name. Hence, we define

3.14 $$f_{1}(y)\overset{\mathrm{def}}{=}v_{1}(y)-y{\hat{u}}_{0}(y).$$

Inputting the form (3.12) into (3.13), leads to two conditions on *f*_1_:

3.15 $$\text{⟦}f_{1}\text{⟧}=\frac{1}{k}\langle \langle \beta f_{1}^{\prime }\rangle \rangle +\frac{1}{k}\langle \langle \beta {\hat{u}}_{0}\rangle \rangle$$

and

3.16 $$-m\mu_{0}^{2}\langle \langle f_{1}\rangle \rangle =\text{⟦}\beta f_{1}^{\prime }\text{⟧}+\text{⟦}\beta {\hat{u}}_{0}\text{⟧}.$$

One should notice, in particular, that no terms involving $U_{1}(x)$ appear in these conditions.

**Remark 3.1.**

For practical computations of the function $v_{1}(y)$, which are required when dealing with specific examples, we can use lemma 2.4 to rewrite the two jump conditions (3.15) and (3.16) as

$$L_{1}[{\text{v}}_{1}]=K_{1}[{\hat{u}}_{0}]\;\text{and}\;L_{2}[{\text{v}}_{1}]=K_{2}[{\hat{u}}_{0}],$$

where

$$\begin{array}{rl} & L_{1}[v_{1}]=v_{1}(0^{+})\mp v_{1}(1^{-})-\frac{1}{2k}(\beta (0^{+})v_{1}^{\prime }(0^{+})\pm \beta (1^{-})v_{1}^{\prime }(1^{-})),\\ & K_{1}[{\hat{u}}_{0}]=\mp {\hat{u}}_{0}(1^{-})\mp \frac{1}{2k}\beta (1^{-}){\hat{u}}_{0}^{\prime }(1^{-}),\\ & L_{2}[v_{1}]=-\frac{m\mu_{0}^{2}}{2}(v_{1}(0^{+})\pm v_{1}(1^{-}))-(\beta (0^{+})v_{1}^{\prime }(0^{+})\mp \beta (1^{-})v_{1}^{\prime }(1^{-})),\\ \;\text{and}\; & K_{2}[{\hat{u}}_{0}]=\mp \frac{m\mu_{0}^{2}}{2}{\hat{u}}_{0}(1^{-})\pm \beta (1^{-}){\hat{u}}_{0}^{\prime }(1^{-}), \end{array}$$

where Notation 2.7 has been used. Note that $L_{1,2}$ are the same operators as those applied to ${\hat{u}}_{0}$ when determining *μ*_0_, though in this case the RHS was 0. Here we have these non-zero $K_{1,2}$ terms.

### (c) Second-order field

We can now collect the terms of order *δ*^2^ in (2.10)–(2.12) to obtain the following equation governing the second-order field *u*_2_:

3.17 $$\frac{\partial }{\partial y}\left(\beta \left(\frac{\partial u_{2}}{\partial y}+\frac{\partial u_{1}}{\partial x}\right)\right)+\mu_{0}^{2}\alpha u_{2}+\beta \frac{\partial^{2}u_{1}}{\partial x\partial y}+\beta \frac{\partial^{2}u_{0}}{\partial x^{2}}+\mu_{2}^{2}\alpha u_{0}=0,$$

subject to the jump conditions

3.18 $$\text{⟦}u_{2}\text{⟧}=\frac{1}{k}\left\langle \left\langle \beta \left(\frac{\partial u_{2}}{\partial y}+\frac{\partial u_{1}}{\partial x}\right)\right\rangle \right\rangle \;\text{and}\;\left[\left[\beta \left(\frac{\partial u_{2}}{\partial y}+\frac{\partial u_{1}}{\partial x}\right)\right]\right]=-m(\mu_{0}^{2}\langle \langle u_{2}\rangle \rangle +\mu_{2}^{2}\langle \langle u_{0}\rangle \rangle ).$$

Similarly to §3b(i), we consider the quantity $\left\langle u_{2}\times \text{(3.1a)}-u_{0}\times (3.17)\right\rangle$. The terms in $\alpha \mu_{0}^{2}u_{0}u_{2}$ cancel out, and, using the fact that

$$u_{2}\frac{\partial }{\partial y}\left(\beta \frac{\partial u_{0}}{\partial y}\right)-u_{0}\frac{\partial }{\partial y}\left(\beta \left(\frac{\partial u_{2}}{\partial y}+\frac{\partial u_{1}}{\partial x}\right)\right)=\frac{\partial }{\partial y}\left(u_{2}\beta \frac{\partial u_{0}}{\partial y}-u_{0}\beta \left(\frac{\partial u_{2}}{\partial y}+\frac{\partial u_{1}}{\partial x}\right)\right)+\beta \frac{\partial u_{0}}{\partial y}\frac{\partial u_{1}}{\partial x},$$

we obtain

3.19 $$\left\langle \frac{\partial }{\partial y}\left(u_{2}\beta \frac{\partial u_{0}}{\partial y}-u_{0}\beta \left(\frac{\partial u_{2}}{\partial y}+\frac{\partial u_{1}}{\partial x}\right)\right)\right\rangle =\left\langle \beta \left(u_{0}\frac{\partial^{2}u_{1}}{\partial x\partial y}-\frac{\partial u_{0}}{\partial y}\frac{\partial u_{1}}{\partial x}+u_{0}\frac{\partial^{2}u_{0}}{\partial x^{2}}\right)\right\rangle +\mu_{2}^{2}\langle \alpha u_{0}^{2}\rangle .$$

Now, note that, by directly using (3.4), (3.12) and (3.14) we can show that

3.20 $$u_{0}\frac{\partial^{2}u_{1}}{\partial x\partial y}-\frac{\partial u_{0}}{\partial y}\frac{\partial u_{1}}{\partial x}+u_{0}\frac{\partial^{2}u_{0}}{\partial x^{2}}=U_{0}(x)U_{0}^{\prime \prime }(x)w_{1}(y),$$

where we define

3.21 $$w_{1}\overset{\mathrm{def}}{=}{\hat{u}}_{0}f_{1}^{\prime }-{\hat{u}}_{0}^{\prime }f_{1}+{\left({\hat{u}}_{0}\right)}^{2}.$$

Moreover, the first bracket of (3.19) can be simplified using note 2.3 and lemma 2.6 and, therefore, using (3.20), (3.19) becomes

3.22 $$-\left[\left[u_{2}\beta \frac{\partial u_{0}}{\partial y}-u_{0}\beta \left(\frac{\partial u_{2}}{\partial y}+\frac{\partial u_{1}}{\partial x}\right)\right]\right]=U_{0}(x)U_{0}^{\prime \prime }(x)\langle \beta w_{1}\rangle +\mu_{2}^{2}\langle \alpha u_{0}^{2}\rangle .$$

**Remark 3.2.**

Inputting (3.14) into (3.21), one shows that $w_{1}={\hat{u}}_{0}v_{1}^{\prime }-{\hat{u}}_{0}^{\prime }v_{1}$, which is the Wronskian associated to the second-order ordinary differential equation (ODE) ${\left(\beta g^{\prime }\right)}^{\prime }+\mu_{0}^{2}\alpha g=0$, and hence satisfies the first-order ODE (*βw*_1_)′ = 0. Moreover, the hypothesis made in note 2.3 regarding potential material properties discontinuities within the interior of the unit cell implies that ${\hat{u}}_{0}$, $\beta {\hat{u}}_{0}^{\prime }$, *u*_1_ and $\beta ((\partial u_{1}/\partial y)+U_{0}^{\prime }(x){\hat{u}}_{0})$ are continuous in *y* on (0, 1). Using the form (3.12) of *u*_1_, this implies that both $v_{1}$ and $\beta v_{1}^{\prime }$ should be continuous on (0, 1), and hence that *βw*_1_ is continuous on (0, 1), which, using the fact that (*βw*_1_)′ = 0, implies that *βw*_1_ is constant on (0, 1). Therefore 〈*βw*_1_〉 = *β*(0^+^) *w*_1_ (0^+^) say, and its computation does not require any integration. Moreover, since ${\hat{u}}_{0}$ and v_1_ are independent, it is clear that 〈*βw*_1_〉 ≠ 0.

As in §3b(i), we can now make use of lemma 2.5 to simplify the left-hand side (LHS) of (3.22):

3.23 $$\begin{array}{rl} -\left[\left[u_{2}\beta \frac{\partial u_{0}}{\partial y}-u_{0}\beta \left(\frac{\partial u_{2}}{\partial y}+\frac{\partial u_{1}}{\partial x}\right)\right]\right] & =-\text{⟦}u_{2}\text{⟧}\left\langle \left\langle \beta \frac{\partial u_{0}}{\partial y}\right\rangle \right\rangle -\langle \langle u_{2}\rangle \rangle \left[\left[\beta \frac{\partial u_{0}}{\partial y}\right]\right]+\text{⟦}u_{0}\text{⟧}\left\langle \left\langle \beta \left(\frac{\partial u_{2}}{\partial y}+\frac{\partial u_{1}}{\partial x}\right)\right\rangle \right\rangle \\ & \;+\langle \langle u_{0}\rangle \rangle \left[\left[\beta \left(\frac{\partial u_{2}}{\partial y}+\frac{\partial u_{1}}{\partial x}\right)\right]\right]\\ & =-k\text{⟦}u_{2}\text{⟧⟦}u_{0}\text{⟧}+m\mu_{0}^{2}\langle \langle u_{2}\rangle \rangle \langle \langle u_{0}\rangle \rangle +k\text{⟦}u_{0}\text{⟧⟦}u_{2}\text{⟧}\\ & \;-m\langle \langle u_{0}\rangle \rangle (\mu_{0}^{2}\langle \langle u_{2}\rangle \rangle +\mu_{2}^{2}\langle \langle u_{0}\rangle \rangle )=-m\mu_{2}^{2}\langle \langle u_{0}{\rangle \rangle }^{2}, \end{array}$$

where the jump conditions (3.1*b*) and (3.18) have been used. Finally, using (3.23) and dividing through by $U_{0}(x)$, (3.22) can be rewritten as

3.24 $$TU_{0}^{\prime \prime }(x)+\mu_{2}^{2}U_{0}(x)=0,\;\text{where}\;T=\frac{\left\langle \beta w_{1}\right\rangle }{\langle \alpha {\left({\hat{u}}_{0}\right)}^{2}\rangle +m{\left\langle \langle {\hat{u}}_{0}\rangle \right\rangle }^{2}},$$

which is the effective equation for $U_{0}$.

### (d) Approximation of the dispersion branches

Note that in (3.24) *T* ≠ 0, but it can be either negative or positive. Since we are looking for standing waves, we seek $\mu_{2}^{2}$ such that $\mu_{2}^{2}/T\geq 0$. Remember that *μ*_2_ is a correction term to the reduced frequency *μ*, such that $\mu^{2}=\mu_{0}^{2}+\delta^{2}\mu_{2}^{2}+o(\delta^{2})$. This means that for each branch of the dispersion diagram (determined by our initial choice of eigenvalue *μ*_0_), we look for a function *μ*_2_ (*κ*) that will lead to an approximation of *μ*(*κ*) at the second order in *δ*, where *κ* is the reduced Bloch wavenumber. In particular, by definition of the FFLW and FFFW cases, we should have

3.25 $$\text{(FFLW) }\mu_{2}(\kappa )\underset{\kappa \to 0}{⟶}0\;\text{and}\;\text{(FFFW)}\;\mu_{2}(\kappa )\underset{\kappa \to \frac{\pi }{\delta }}{⟶}0.$$

In order for our asymptotic representation (2.9) to be compatible with the fact that *u*_*δ*_ should satisfy the Bloch–Floquet conditions (2.8), it is enough to impose that all the *u*_*j*_ (*x*, *x*/*δ*) should also satisfy these conditions. For *j* = 0, this means that

3.26 $$U_{0}(x+\delta ){\hat{u}}_{0}\left(\frac{x}{\delta }+1\right)=U_{0}(x){\hat{u}}_{0}\left(\frac{x}{\delta }\right){\text{e}}^{\text{i}\kappa \delta }.$$

Hence, due to the fact that ${\hat{u}}_{0}$ is periodic (FFLW) or antiperiodic (FFFW), we can cancel out the terms ${\hat{u}}_{0}$ in (3.26) to get

3.27 $$U_{0}(x+\delta )=\pm U_{0}(x)\;{\text{e}}^{\text{i}\kappa \delta },$$

where, in (3.27) and below, Notation 2.7 is being used. The second Bloch–Floquet condition in (2.8), combined with (3.27), implies that

3.28 $$U_{0}^{\prime }(x+\delta )=\pm U_{0}^{\prime }(x)\;{\text{e}}^{\text{i}\kappa \delta }.$$

Because $U_{0}$ is a solution to (3.24), it can be written $U_{0}(x)=A\;{\text{e}}^{\text{i}\sqrt{\mu_{2}^{2}/T}x}+B\;{\text{e}}^{-\text{i}\sqrt{\mu_{2}^{2}/T}x}$ for some constants *A* and *B*. The Bloch–Floquet conditions (3.27) and (3.28) lead to

3.29 $$A\left(1\mp {\text{e}}^{\text{i}\delta \left(\sqrt{\mu_{2}^{2}/T}-\kappa \right)}\right)=0\;\text{and}\;B\left(1\mp {\text{e}}^{-\text{i}\delta \left(\sqrt{\mu_{2}^{2}/T}+\kappa \right)}\right)=0.$$

Since *κ* is restricted to (0, *π*/*δ*), i.e. to the first Brillouin zone in the dispersion diagram, and since it is assumed that $\mu_{2}^{2}/T\geq 0$, (3.29) implies that, using (3.3) , we have

3.30 $$\sqrt{\frac{\mu_{2}^{2}}{T}}=\tilde{\kappa }\;\text{and}\;U_{0}(x)={\text{e}}^{\pm \text{i}\tilde{\kappa }x},$$

which gives the following approximation for the reduced frequency *μ*(*κ*)

3.31 $$\mu^{2}=\mu_{0}^{2}+T{\left(\tilde{\kappa }\delta \right)}^{2}+o({\tilde{\kappa }}^{2}\delta^{2})\;\text{or equivalently}\;\mu =\mu_{0}+\frac{T}{2\mu_{0}}{\left(\tilde{\kappa }\delta \right)}^{2}+o({\tilde{\kappa }}^{2}\delta^{2}).$$

The non-dimensional wave field is approximated by

3.32 $$u_{\delta }(x)=\underset{u_{0}(x,x/\delta )}{\underbrace{U_{0}(x){\hat{u}}_{0}\left(\frac{x}{\delta }\right)}}+O(\tilde{\kappa }\delta ).$$

Note that in §6, we will find it more convenient to test the validity of (3.32) when it is written in terms of the variable *y* as follows

3.33 $$u_{\delta }(\delta y)=\underset{u_{0}(\delta y,y)}{\underbrace{U_{0}(\delta y){\hat{u}}_{0}(y)}}+O(\tilde{\kappa }\delta ).$$

Hence, as anticipated, using (2.4), our results can be summarized in dimensional form by (1.2) and (1.3), where the parameter T and the leading-order wave field $U_{h}^{\left(0\right)}$ are given by

$$T=\frac{{\left(c^{⋆}\right)}^{2}}{h^{2}}T,\;\omega_{0}=\frac{c^{⋆}\mu_{0}}{h}\;\text{and}\;U_{h}^{\left(0\right)}(X)={\text{e}}^{\pm \text{i}\tilde{k}X}{\hat{u}}_{0}\left(\frac{X}{h}\right).$$

## 4. The case of a double eigenvalue *μ*_0_

### (a) A new form for *u*_0_

In order to write (3.4), we assumed that *μ*_0_ was a simple eigenvalue. If instead we assume that *μ*_0_ has multiplicity 2 say, then we write

4.1 $$u_{0}(x,y)=\underset{u_{0}^{\left(1\right)}(x,y)}{\underbrace{U_{0}^{\left(1\right)}(x){\hat{u}}_{0}^{\left(1\right)}(y)}}\;+\;\underset{u_{0}^{\left(2\right)}(x,y)}{\underbrace{U_{0}^{\left(2\right)}(x){\hat{u}}_{0}^{\left(2\right)}(y)}}\;,$$

where ${\hat{u}}_{0}^{\left(1\right)}(y)$ and ${\hat{u}}_{0}^{\left(2\right)}(y)$ are two independent eigenfunctions associated with the double eigenvalue *μ*_0_ and $U_{0}^{\left(1\right)}(x)$ and $U_{0}^{\left(2\right)}(x)$ are some functions of *x* to be determined. Note that both ${\hat{u}}_{0}^{\left(1\right)}(y)$ and ${\hat{u}}_{0}^{\left(2\right)}(y)$ satisfy (3.1*a*)–(3.1*b*). In what follows, for any *j* ∈ {1, 2}, we will use the notation (3.1*a*^(*j*)^) and (3.1*b*^(*j*)^), to specify that we consider (3.1*a*)–(3.1*b*) as applied to ${\hat{u}}_{0}^{\left(j\right)}(y)$.

### (b) In this case, we cannot conclude that *μ*_1_ = 0

We will now apply the same methodology as in §3b(i) and consider the quantity $\left\langle u_{1}\times (3.1{\text{a}}^{\left(1\right)})-u_{0}^{\left(1\right)}\times (3.5)\right\rangle$. The exact same reasoning leads to the counterpart to (3.7):

4.2 $$\left\langle \frac{\partial }{\partial y}\left(u_{1}\beta \frac{\partial u_{0}^{\left(1\right)}}{\partial y}-u_{0}^{\left(1\right)}\beta \left(\frac{\partial u_{1}}{\partial y}+\frac{\partial u_{0}}{\partial x}\right)\right)\right\rangle =\left\langle \beta \left(u_{0}^{\left(1\right)}\frac{\partial^{2}u_{0}}{\partial x\partial y}-\frac{\partial u_{0}}{\partial x}\frac{\partial u_{0}^{\left(1\right)}}{\partial y}\right)\right\rangle +\mu_{1}^{2}\langle \alpha u_{0}^{\left(1\right)}u_{0}\rangle .$$

The only difference being that this time, the first bracket in the RHS of (4.2) is not zero. Instead, it can be shown directly using (4.1) that

4.3 $$u_{0}^{\left(1\right)}\frac{\partial^{2}u_{0}}{\partial x\partial y}-\frac{\partial u_{0}}{\partial x}\frac{\partial u_{0}^{\left(1\right)}}{\partial y}=U_{0}^{\left(1\right)}(x)U_{0}^{(2)\prime }(x)w_{0}(y),$$

where $w_{0}$ is the Wronskian defined by

4.4 $$w_{0}(y)={\hat{u}}_{0}^{\left(1\right)}(y){\hat{u}}_{0}^{(2)\prime }(y)-{\hat{u}}_{0}^{(1)\prime }(y){\hat{u}}_{0}^{\left(2\right)}(y).$$

The same methodology to simplify the LHS bracket in (4.2) as that used in §3b(i) can be used: first use lemma 2.6 to reduce the average bracket to a jump bracket, then use lemma 2.5 to decompose the jump bracket into four simpler jump/mean brackets that can be computed using the jump conditions (3.1*b*^(1)^) and (3.6) to obtain

4.5 $$-m\mu_{1}^{2}\langle \langle u_{0}^{\left(1\right)}\rangle \rangle \langle \langle u_{0}\rangle \rangle =U_{0}^{\left(1\right)}(x)U_{0}^{(2)\prime }(x)\langle \beta w_{0}\rangle +\mu_{1}^{2}\langle \alpha u_{0}^{\left(1\right)}u_{0}\rangle ,$$

which, upon regrouping the terms, dividing through by $U_{0}^{\left(1\right)}(x)$ and using (4.1), can be rewritten

4.6 $$\langle \beta w_{0}\rangle U_{0}^{(2)\prime }(x)=-\mu_{1}^{2}(\{mB_{1}^{2}+C_{1}\}U_{0}^{\left(1\right)}(x)+\{mB_{1}B_{2}+D\}U_{0}^{\left(2\right)}(x)),$$

where we have defined

4.7 $$B_{1}=\langle \langle {\hat{u}}_{0}^{\left(1\right)}\rangle \rangle ,\;B_{2}=\langle \langle {\hat{u}}_{0}^{\left(2\right)}\rangle \rangle ,\;C_{1}=\langle \alpha {\left({\hat{u}}_{0}^{\left(1\right)}\right)}^{2}\rangle ,\;C_{2}=\langle \alpha {\left({\hat{u}}_{0}^{\left(2\right)}\right)}^{2}\rangle ,\;D=\langle \alpha {\hat{u}}_{0}^{\left(1\right)}{\hat{u}}_{0}^{\left(2\right)}\rangle .$$

In the exact same way, we consider the quantity $\left\langle u_{1}\times (3.1{\text{a}}^{\left(2\right)})-u_{0}^{\left(2\right)}\times (3.5)\right\rangle$ to obtain

4.8 $$\langle \beta w_{0}\rangle U_{0}^{(1)\prime }(x)=\mu_{1}^{2}(\{mB_{1}B_{2}+D\}U_{0}^{\left(1\right)}(x)+\{mB_{2}^{2}+C_{2}\}U_{0}^{\left(2\right)}(x)).$$

Note that $w_{0}$ is the Wronskian determinant associated with (3.1*a*), and $\beta w_{0}$ can be shown to be continuous on the unit cell, hence we conclude that $\beta w_{0}$ is actually constant, and hence we can see that $\left\langle \beta w_{0}\rangle =\beta (0^{+})w_{0}(0^{+}\right)$. Since ${\hat{u}}_{0}^{\left(1\right)}$ and ${\hat{u}}_{0}^{\left(2\right)}$ are linearly independent, $w_{0}$ (being the associated Wronskian) is also non-zero and in this case, we cannot conclude that *μ*_1_ = 0.

### (c) Solving a first-order ODE system to obtain *μ*_1_

Upon introducing the function vector $U={\left(U_{0}^{\left(1\right)},U_{0}^{\left(2\right)}\right)}^{T}$, the two equations (4.6) and (4.8) can be recast as the first-order ODE system

4.9 $$U^{\prime }(x)=\frac{\mu_{1}^{2}}{\left\langle \beta w_{0}\right\rangle }NU(x),\;\text{where}\;N=\left(\begin{array}{cc} mB_{1}B_{2}+D & mB_{2}^{2}+C_{2}\\ -(mB_{1}^{2}+C_{1}) & -(mB_{1}B_{2}+D) \end{array}\right).$$

The two eigenvalues *λ*_1,2_ of N are given by

4.10 $$\lambda_{j}=i{\left(-1\right)}^{j}\sqrt{(mB_{1}^{2}+C_{1})(mB_{2}^{2}+C_{2})-{\left(mB_{1}B_{2}+D\right)}^{2}},$$

where, by the Cauchy–Schwarz inequality associated with the inner product (3.2), the quantity inside the square root is positive. The associated eigenvectors are given by

4.11 $$U_{\lambda_{1}}={\left(\frac{-(mB_{1}B_{2}+D)+\lambda_{2}}{mB_{1}^{2}+C_{1}},1\right)}^{T}\;\text{and}\;U_{\lambda_{2}}={\left(\frac{-(mB_{1}B_{2}+D)+\lambda_{1}}{mB_{1}^{2}+C_{1}},1\right)}^{T}$$

and hence, upon introducing *T*_*d*_ to be

4.12 $$T_{d}=\frac{\left\langle \beta w_{0}\right\rangle }{\sqrt{(mB_{1}^{2}+C_{1})(mB_{2}^{2}+C_{2})-{\left(mB_{1}B_{2}+D\right)}^{2}}},$$

the solution to (4.9) can be written as

4.13 $$U(x)=c_{1}U_{\lambda_{1}}\;{\text{e}}^{(\mu_{1}^{2}/\left\langle \beta w_{0}\right\rangle )\lambda_{1}x}+c_{2}U_{\lambda_{2}}\;{\text{e}}^{(\mu_{1}^{2}/\left\langle \beta w_{0}\right\rangle )\lambda_{2}x}=c_{1}U_{\lambda_{1}}\;{\text{e}}^{-\text{i}(\mu_{1}^{2}/T_{d})x}+c_{2}U_{\lambda_{2}}\;{\text{e}}^{\text{i}(\mu_{1}^{2}/T_{d})x},$$

for some constants $c_{1,2}$. At this stage, we need to remember the Bloch–Floquet conditions (2.8), which, when applied to *u*_0_, imply that

$${\hat{u}}_{0}^{\left(1\right)}(y)\{\pm U_{0}^{\left(1\right)}(x+\delta )-{\text{e}}^{\text{i}\kappa \delta }U_{0}^{\left(1\right)}(x)\}+{\hat{u}}_{0}^{\left(2\right)}(y)\{\pm U_{0}^{\left(2\right)}(x+\delta )-{\text{e}}^{\text{i}\kappa \delta }U_{0}^{\left(2\right)}(x)\}=0,$$

where Notation 2.7 has been used. Since ${\hat{u}}_{0}^{\left(1\right)}$ and ${\hat{u}}_{0}^{\left(2\right)}$ are linearly independent, this implies that $\pm U(x+\delta )={\text{e}}^{\text{i}\kappa \delta }U(x)$. Applying this condition to (4.13), leads to the value of $\mu_{1}^{2}$ as follows

4.14 $$\delta \mu_{1}^{2}=+T_{d}\tilde{\kappa }\delta \;\text{or}\;\delta \mu_{1}^{2}=-T_{d}\tilde{\kappa }\delta .$$

Hence, since $\mu^{2}=\mu_{0}^{2}+\delta \mu_{1}^{2}+o(\delta )$, we obtain the linear approximations

4.15 $$\mu =\mu_{0}\pm \frac{T_{d}}{2\mu_{0}}(\tilde{\kappa }\delta )+o(\tilde{\kappa }\delta ).$$

Here, the symbol ± should not be understood as per Notation 2.7, but as two different slopes, so that, near each double eigenvalue *μ*_0_ of the dispersion diagram, we have two linear approximations with opposite slopes emerging from *μ*_0_. Such behaviour of the dispersion diagram, is that of so-called Dirac points.

## 5. The case of two nearby eigenvalues

In what has been done above, there is no uniform transition from the simple eigenvalue case to the double eigenvalue case. As will be seen in the examples of §6, when two simple eigenvalues are close to each other, the agreement between the dispersion diagram and the asymptotic of §3 is somewhat short-lived. In order to remedy this issue, following some ideas developed in [7,28], we will derive an asymptotic expansion for two nearby eigenvalues.

Let us assume that we have two nearby eigenvalues $\mu_{0}^{\left(1\right)}$ and $\mu_{0}^{\left(2\right)}$ with their associated eigenfunctions ${\hat{u}}_{0}^{\left(1\right)}(y)$ and ${\hat{u}}_{0}^{\left(2\right)}(y)$ that solve (3.1*a*)–(3.1*b*) and such that $\mu_{0}^{\left(1\right)}<\mu_{0}^{\left(2\right)}$. Since we are seeking an approximation that remains valid when two eigenvalues merge into one, we assume that $\mu_{0}^{\left(1,2\right)}$ both belong to the same side of the dispersion diagram, i.e. they are either both FFLW or both FFFW. Their proximity is characterized by a positive parameter γ=O(1) defined by

5.1 $$\delta \gamma ={\left(\mu_{0}^{\left(2\right)}\right)}^{2}-{\left(\mu_{0}^{\left(1\right)}\right)}^{2}.$$

In the vicinity of these eigenvalues we seek expansions of the form (2.9), in which we choose $\mu_{0}=\mu_{0}^{\left(1\right)}$. In order to consider the competing nature of the two eigenvalues, we seek *u*_0_ in the form (4.1), introducing the functions $U_{0}^{\left(1,2\right)}(x)$ and $u_{0}^{\left(1,2\right)}(x,y)$ accordingly. With such a choice of *u*_0_, one can show directly that we have

5.2 $$\frac{\partial }{\partial y}\left(\beta \frac{\partial u_{0}}{\partial y}\right)+{\left(\mu_{0}^{\left(1\right)}\right)}^{2}\alpha u_{0}=-\delta \gamma \alpha u_{0}^{\left(2\right)}\;\text{with}\;\left\{ \begin{array}{l} \text{⟦}u_{0}\text{⟧}=\frac{1}{k}\langle \langle \beta \frac{\partial u_{0}}{\partial y}\rangle \rangle ,\\ {}[[\beta \frac{\partial u_{0}}{\partial y}]]=-m{\left(\mu_{0}^{\left(1\right)}\right)}^{2}\langle \langle u_{0}\rangle \rangle -m\delta \gamma \langle \langle u_{0}^{\left(2\right)}\rangle \rangle . \end{array}\right.$$

The terms involving *δ* in the RHSs of (5.2) should be considered when collecting the terms of order *δ* in (2.10)–(2.12) to obtain the equation governing *u*_1_:

5.3 $$\frac{\partial }{\partial y}\left(\beta \left(\frac{\partial u_{1}}{\partial y}+\frac{\partial u_{0}}{\partial x}\right)\right)+\beta \frac{\partial^{2}u_{0}}{\partial x\partial y}+\alpha ({\left(\mu_{0}^{\left(1\right)}\right)}^{2}u_{1}+\mu_{1}^{2}u_{0}-\gamma u_{0}^{\left(2\right)})=0,$$

and the associated jump conditions

5.4 $$\begin{array}{r} \text{⟦}u_{1}\text{⟧}=\frac{1}{k}\left\langle \left\langle \beta \left(\frac{\partial u_{0}}{\partial x}+\frac{\partial u_{1}}{\partial y}\right)\right\rangle \right\rangle \;\text{and}\;\left[\left[\beta \left(\frac{\partial u_{0}}{\partial x}+\frac{\partial u_{1}}{\partial y}\right)\right]\right]=-m({\left(\mu_{0}^{\left(1\right)}\right)}^{2}\langle \langle u_{1}\rangle \rangle +\mu_{1}^{2}\langle \langle u_{0}\rangle \rangle -\gamma \langle \langle u_{0}^{\left(2\right)}\rangle \rangle ). \end{array}$$

After following the exact same strategy as in §4, consider first the term $\left\langle u_{1}\times (3.1a^{\left(1\right)})-u_{0}^{\left(1\right)}\times (5.3)\right\rangle$, and then the term $\left\langle u_{1}\times (3.1a^{\left(2\right)})-u_{0}^{\left(2\right)}\times (5.3)\right\rangle$, to obtain two equations that can be recast in the first-order ODE system

5.5 $$U^{\prime }(x)=\frac{\mu_{1}^{2}}{\left\langle \beta w_{0}\right\rangle }N_{\gamma }U(x).$$

Here we used $U={\left(U_{0}^{\left(1\right)},U_{0}^{\left(2\right)}\right)}^{T}$, the function w_0_ is defined as in (4.4) and $N_{\gamma }$ is given by

5.6 $$N_{\gamma }=\left(\begin{array}{cc} mB_{1}B_{2}+D & \left(1-\frac{\gamma }{\mu_{1}^{2}})(mB_{2}^{2}+C_{2}\right)\\ -(mB_{1}^{2}+C_{1}) & -(1-\frac{\gamma }{\mu_{1}^{2}})(mB_{1}B_{2}+D) \end{array}\right)=N+\frac{\gamma }{\mu_{1}^{2}}\left(\begin{array}{cc} 0 & -mB_{2}^{2}-C_{2}\\ 0 & mB_{1}B_{2}+D \end{array}\right),$$

the parameters *B*_1,2_, *C*_1,2_ and *D* being defined as in (4.7). Note that when taking *γ* → 0 in (5.5), we recover exactly (4.9), showing the consistency of our approach. Note that when deriving the second line of (5.5), we neglect the term $\delta \gamma \langle u_{1},{\hat{u}}_{0}^{\left(2\right)}\rangle$ that occurs in the process since it is of order *δ*. The eigenvalues $\lambda_{1,2}^{\left(\gamma \right)}$ of $N_{\gamma }$ can be found to be given by

$$\lambda_{j}^{\left(\gamma \right)}=\frac{(mB_{1}B_{2}+D)\gamma +{\left(-1\right)}^{j}\sqrt{{\left(mB_{1}B_{2}+D\right)}^{2}{\left(\gamma -2\mu_{1}^{2}\right)}^{2}+4(mB_{1}^{2}+C_{1})(mB_{2}^{2}+C_{2})\mu_{1}^{2}(\gamma -\mu_{1}^{2})}}{2\mu_{1}^{2}},$$

while the associated eigenvectors are

5.7 $$U_{\lambda_{1}^{\left(\gamma \right)}}={\left(\frac{-(mB_{1}B_{2}+D)+\lambda_{2}^{\left(\gamma \right)}}{\left(mB_{1}^{2}+C_{1}\right)},1\right)}^{T}\;\text{and}\;U_{\lambda_{2}^{\left(\gamma \right)}}={\left(\frac{-(mB_{1}B_{2}+D)+\lambda_{1}^{\left(\gamma \right)}}{\left(mB_{1}^{2}+C_{1}\right)},1\right)}^{T}.$$

We can hence follow what we have done in §4c, and use the Bloch–Floquet conditions to obtain

$$(\mathrm{FFLW}):\frac{\mu_{1}^{2}\lambda_{j}^{\left(\gamma \right)}}{\left\langle \beta w_{0}\right\rangle }=i\kappa \;\text{and}\;(\mathrm{FFFW}):\frac{\mu_{1}^{2}\lambda_{j}^{\left(\gamma \right)}}{\left\langle \beta w_{0}\right\rangle }=-i\left(\frac{\pi }{\delta }-\kappa \right),$$

which are implicit relationships between $\mu_{1}^{2}$ and *κ*. Fortunately, these can be inverted exactly to obtain

5.8 $$\mu_{1}^{2}=\frac{1}{2}\left(\gamma \mp \sqrt{4T_{d}^{2}{\tilde{\kappa }}^{2}+4iT_{d}^{2}\frac{\left(mB_{1}B_{2}+D\right)}{\left\langle \beta w_{0}\right\rangle }\tilde{\kappa }\gamma +\gamma^{2}}\right),$$

where (3.3) has been used, and where *T*_*d*_ is defined as in (4.12). Here ∓ should not be understood as per Notation 2.7, but as two different branches. One issue with (5.8) is that, in its current form, it implies that $\mu_{1}^{2}$ is actually complex. However, this issue is settled by realizing that upon using the inner product defined in (3.2), we can write

$$mB_{1}B_{2}+D=\langle {\hat{u}}_{0}^{\left(1\right)},{\hat{u}}_{0}^{\left(2\right)}\rangle .$$

Hence, since ${\hat{u}}_{0}^{\left(1,2\right)}$ correspond to two different eigenvalues, they are orthogonal and we get $mB_{1}B_{2}+D=0$. Therefore, the complex part of (5.8) disappears and it simplifies to

5.9 $$\delta \mu_{1}^{2}=\frac{1}{2}\left(\delta \gamma \mp \sqrt{4T_{d}^{2}{\left(\tilde{\kappa }\delta \right)}^{2}+{\left(\delta \gamma \right)}^{2}}\right)\;\text{and}\;\mu^{2}={\left(\mu_{0}^{\left(1\right)}\right)}^{2}+\delta \mu_{1}^{2}+o(\delta ).$$

In the limit *γ* → ∞ or $\tilde{\kappa }\to 0$, $\mu_{1}^{2}$ behaves like $\mu_{1}^{2}\to 0$ or $\mu_{1}^{2}∼\gamma$, depending on the sign chosen in (5.8). This allows us to conclude that the − sign corresponds to the branch emanating from $\mu_{0}^{\left(1\right)}$, while the + sign corresponds to the branch emanating from $\mu_{0}^{\left(2\right)}$. There are two other interesting limits to consider.

The first is to see what happens when two eigenvalues are merging, i.e. we fix $\tilde{\kappa }\delta$ and let *δγ* → 0. In this case (5.9) simplifies to $\mu^{2}\approx {\left(\mu_{0}^{\left(1\right)}\right)}^{2}+(\delta \gamma /2)\mp T_{d}\tilde{\kappa }\delta$, which, when plotted against $\tilde{\kappa }\delta$ are two straight lines with opposite slopes emanating from a point between the two nearby eigenvalues. When the two eigenvalues merge (i.e. *δγ* = 0), we recover exactly the double eigenvalue approximation (4.14).

The second is to see how well (5.9) approximates the dispersion diagram at the edges of the Brillouin zone for a given *δγ* ≠ 0. So we fix *δγ* and let $\tilde{\kappa }\delta \to 0$. In this case, (5.9) simplifies to

$$\mu^{2}\approx {\left(\mu_{0}^{\left(1\right)}\right)}^{2}+\frac{1}{2}(\delta \gamma \mp \delta \gamma )\mp \frac{T_{d}^{2}}{\delta \gamma }{\left(\tilde{\kappa }\delta \right)}^{2}.$$

Hence, for the lower branch it becomes $\mu^{2}\approx {\left(\mu_{0}^{\left(1\right)}\right)}^{2}-(T_{d}^{2}/\delta \gamma ){\left(\tilde{\kappa }\delta \right)}^{2}$, while for the upper branch, it reads $\mu^{2}\approx {\left(\mu_{0}^{\left(2\right)}\right)}^{2}+(T_{d}^{2}/\delta \gamma ){\left(\tilde{\kappa }\delta \right)}^{2}$. This approximation resembles (3.31), but with an incorrect quadratic coefficient (since in general $\mp (T_{d}^{2}/\delta \gamma )\neq T$), so it is only a first-order approximation, slightly less precise than the simple eigenvalue approximation.

Hence, (5.9) is a *uniform approximation*, in the sense that it is valid for both simple and double eigenvalues. Moreover, we will see in the next section that using (5.9) leads to a much longer-lived fit to the exact dispersion diagram than the simple eigenvalue method.

## 6. Examples and numerical experiments

The theory developed above has the advantage to be valid for any spatially varying periodic material properties, even in cases when the dispersion diagram cannot be obtained analytically or is computationally intricate to obtain. However, in order to validate the method, we now consider two simpler examples, for which the dispersion diagram can be obtained directly by the Bloch–Floquet analysis.

### (a) Monolayer

**Figure 2.**
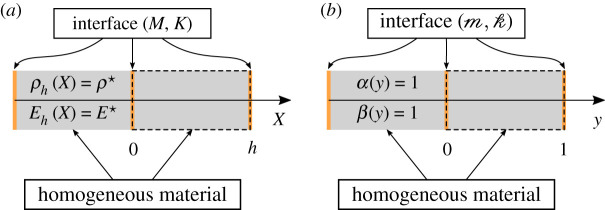
Geometry of the monolayer problem in the physical (*a*) and non-dimensional (*b*) settings. (Online version in colour.)

The simplest example that can be considered is the case of a monolayer material with imperfect interface. By this we mean that the density and Young’s modulus are constant, so that $\rho_{h}(X)=\rho^{⋆}$ and $E_{h}(X)=E^{⋆}$. This implies that *α* = *β* = 1. The geometry of the physical problem is represented in figure 2.

The Bloch–Floquet analysis (see appendix B) gives the following dispersion relation

6.1 $$\cos(\kappa \delta )=\frac{1}{1+\frac{m\mu^{2}}{4k}}\left[\left(1-\frac{m\mu^{2}}{4k}\right)\cos(\mu )-\frac{1}{2}\left(\frac{\mu }{k}+m\mu \right)\sin(\mu )\right].$$

**Figure 3.**
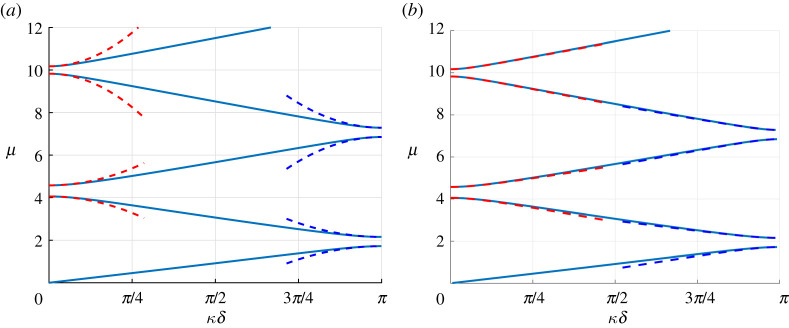
Dispersion diagram for the monolayer with k=1 and m=0.5. The red (resp. blue) dashed lines are the periodic (resp. antiperiodic) second-order approximations (3.31) using the computed values of *T* (*a*) and nearby eigenvalue approximations (5.9) using the computed values of *T*_*d*_ and *δγ* (*b*). (Online version in colour.)

The dispersion diagram classically displays band gaps as can be seen in figure 3. We will now apply the high-frequency homogenization technique to derive an analytical approximation to the higher branches of the diagram and to the associated wave fields.

In the case of a single eigenvalue, using (3.1), we find that ${\hat{u}}_{0}$ can be written as ${\hat{u}}_{0}=A\cos(\mu_{0}y)+B\sin(\mu_{0}y)$ for some constants *A* and *B*, and, using lemma 2.4, it is subject to the jump conditions

6.2 $$\left\{ \begin{array}{rl} & {\hat{u}}_{0}(0^{+})\mp {\hat{u}}_{0}(1^{-})=\frac{1}{2k}({\hat{u}}_{0}^{\prime }(0^{+})\pm {\hat{u}}_{0}^{\prime }(1^{-})),\\ & {\hat{u}}_{0}^{\prime }(0^{+})\mp {\hat{u}}_{0}^{\prime }(1^{-})=\frac{-m\mu_{0}^{2}}{2}({\hat{u}}_{0}(0^{+})\pm {\hat{u}}_{0}(1^{-})), \end{array}\right.$$

where here and throughout the section, Notation 2.7 is being used. This leads to the relation

6.3 $$M^{\mathrm{mo}}{\left(A,B\right)}^{T}={\left(0,0\right)}^{T},$$

where the 2 × 2 matrix $M^{\mathrm{mo}}=(M_{ij}^{\mathrm{mo}})$ is given by

$$M^{\mathrm{mo}}=\left(\begin{array}{cc} 1\mp \cos(\mu_{0})\pm \frac{1}{2k}\mu_{0}\sin(\mu_{0}) & \mp \sin(\mu_{0})-\frac{\mu_{0}}{2k}(1\pm \cos(\mu_{0}))\\ \mp \mu_{0}\sin(\mu_{0})-\frac{m\mu_{0}^{2}}{2}(1\pm \cos(\mu_{0})) & -\mu_{0}(1\mp \cos(\mu_{0}))\mp \frac{m\mu_{0}^{2}}{2}\sin(\mu_{0}) \end{array}\right).$$

The only way for non-trivial solutions to (6.3) to exist is for its determinant to be zero, which after some algebraic manipulations leads to a dispersion relation of the form

6.4 $$D^{\mathrm{mo}}(\mu_{0};m,k)=2(1\mp \cos(\mu_{0}))+\frac{m}{2k}\mu_{0}^{2}(1\pm \cos(\mu_{0}))\pm \mu_{0}\sin(\mu_{0})\left(m+\frac{1}{k}\right)=0,$$

where the fact that *μ*_0_ ≠ 0 has been used (we are not interested here in the low frequency limit). In practice, when calculating *μ*_0_ and reconstructing ${\hat{u}}_{0}$, it can be useful to note that

6.5 $$\begin{array}{r} M_{22}^{\mathrm{mo}}=\frac{\pm \sin(\mu_{0})}{1\pm \cos(\mu_{0})}M_{21}^{\mathrm{mo}},\;M_{12}^{\mathrm{mo}}=\frac{1\pm \cos(\mu_{0})}{\mp \sin(\mu_{0})}M_{11}^{\mathrm{mo}},\;M_{11}^{\mathrm{mo}}M_{21}^{\mathrm{mo}}=\mp \frac{\sin(\mu_{0})}{2}D^{\mathrm{mo}}(\mu_{0};m,k), \end{array}$$

so that *μ*_0_ is either a zero of $M_{11}^{\mathrm{mo}}$ or $M_{21}^{\mathrm{mo}}$, and in the former (resp. latter) case, the top (resp. bottom) line of $M^{\mathrm{mo}}$ is zero (it can be shown that sin (*μ*_0_) ≠ 0). The computed eigenvalues coincide with the edges of the Brillouin zone of the dispersion diagram of figure 3. To obtain ${\hat{u}}_{0}$, we set *A* = 1, so that ${\hat{u}}_{0}(0^{+})=1$, and compute *B* using the first (resp. second) line in (6.3) if $M_{11}^{\mathrm{mo}}\neq 0$ (resp. $M_{21}^{\mathrm{mo}}\neq 0$).

Because we are ultimately interested in the value of *T* in (3.31), we need to calculate 〈*βw*_1_〉 in (3.24), and hence $v_{1}$ on the interval (0, 1). We do this using the fact that it satisfies the same second-order equation (3.1*a*) as ${\hat{u}}_{0}$ and can hence be written $v_{1}(y)=C\cos(\mu_{0}y)+D\sin(\mu_{0}y)$, for some constants *C* and *D*, which, using remark 3.1, can be found by solving $M^{\mathrm{mo}}{\left(C,D\right)}^{T}=b^{\mathrm{mo}},$ where

6.6 $$b^{\mathrm{mo}}=\left(\begin{array}{c} \mp {\hat{u}}_{0}(1^{-})\mp \frac{1}{2k}{\hat{u}}_{0}^{\prime }(1^{-})\\ \mp \frac{m\mu_{0}^{2}}{2}{\hat{u}}_{0}(1^{-})\pm {\hat{u}}_{0}^{\prime }(1^{-}) \end{array}\right).$$

Since $M^{\mathrm{mo}}$ is singular, (6.6) does not have a unique solution so we set *C* = 1 say and use the non-trivial line of the system to determine *D*. This works well since it can be shown that $b^{\mathrm{mo}}$ is such that $b_{j}^{\mathrm{mo}}=0$ whenever $M_{j,1}=0$.

**Figure 4.**
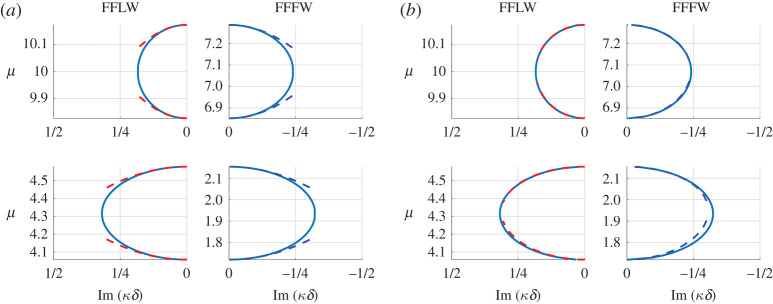
Imaginary part of *κδ* in the band gaps of the monolayer dispersion diagram for k=1 and m=0.5. The red (resp. blue) dashed lines are the periodic (resp. antiperiodic) second-order approximations (3.31) using the computed values of *T* (*a*) and nearby eigenvalue approximations (5.9) using the computed values of *T*_*d*_ and *δγ* (*b*). (Online version in colour.)

Once v_1_ is found, the resulting value of *T* is directly obtained using (3.24). Note that, in this simple case, no numerical integration is required and calculations can be performed analytically. The resulting second-order approximations $\mu \approx \mu_{0}+(T/2\mu_{0}){\left(\tilde{\kappa }\delta \right)}^{2}$ are superposed to the dispersion diagram in figure 3*a*, and one can see that they approximate the branches well in the vicinity of the edges of the Brillouin zone. One should also note that, as seen in figure 4 (left), this approximation remains valid within the band gaps, where *κδ* is complex and is such that Re(κ~δ)=0. This is expected since in these cases, ${\left(\tilde{\kappa }\delta \right)}^{2}$ remains real. Having computed all the eigenvalues and eigenfunctions, it is now straightforward to compute *T*_*d*_ as per (4.12) and *δγ* as per (5.1), where the pairs of eigenvalues are chosen naturally according to the dispersion diagram (first-second) and (third-fourth) in both the FFLW and FFFW cases. Hence we can evaluate the nearby eigenvalue approximation (5.9) derived in §5. It is displayed in figure 3*b*, and as can clearly be seen, even if this approximation is only first-order in the vicinity of the edges of the Brillouin zone, its agreement with the dispersion diagram is much longer lived than that of the simple eigenvalue approximation. Similar observations are true within the band gaps as can be seen in figure 4*b*.

We will now investigate the accuracy of the zeroth-order field approximation obtained in the simple eigenvalue case. Using the Bloch–Floquet analysis (see appendix B), we can have access to the exact standing wave field *u*_*δ*_ (*x*) = *u*_*δ*_ (*δy*), and, to be compatible with the asymptotic expansion (2.9), we normalize it such that *u*_*δ*_ (0^+^) = *u*_0_ (0, 0^+^). Note that because of (2.7) and (3.33), the difference between the exact and approximated field can be written as

6.7 $$u_{\delta }(x)-u_{0}\left(x,\frac{x}{\delta }\right)=u_{\delta }(\delta y)-u_{0}(\delta y,y)=\left\{ \begin{array}{ll} {\text{e}}^{\text{i}\kappa \delta y}(u_{\delta }(\delta y)-{\hat{u}}_{0}(y)) & \text{(FFLW)},\\ {\text{e}}^{\text{i}\kappa \delta y}(u_{\delta }(\delta y)-{\text{e}}^{-\text{i}\pi y}{\hat{u}}_{0}(y)) & \text{(FFFW)}. \end{array}\right.$$

**Figure 5.**
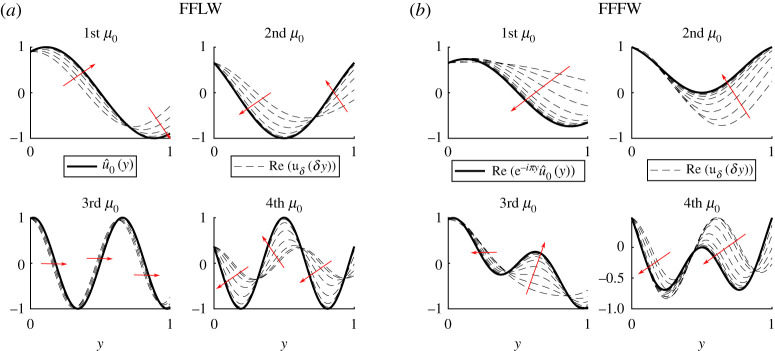
Illustration of the convergence of the method by comparing ${\hat{u}}_{0}$ and $u_{\delta }(\delta y)$ in the FFLW (*a*) and the FFFW (*b*) cases. We plotted $u_{\delta }$ for 20 values of $\tilde{\kappa }\delta$ equidistributed in the log scale between 10^−5^ and 1. The red arrows indicate how $u_{\delta }(\delta y)$ is changing as $\tilde{\kappa }\delta \to 0$. (Online version in colour.)

To illustrate the validity of our approximation, we hence compare $u_{\delta }(\delta y)$ and ${\hat{u}}_{0}(y)$ for various values of *κδ* in figure 5, showing, as expected, that as $\tilde{\kappa }\delta$ gets smaller the zeroth-order field is a good approximation to the exact field.

**Figure 6.**
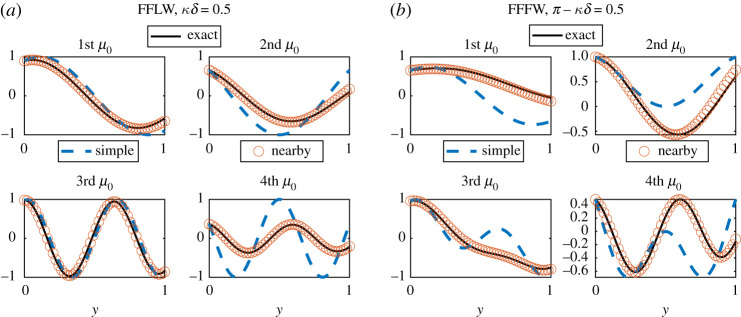
Superposition of real parts of the exact ($u_{\delta }$), simple eigenvalue zeroth-order (${\hat{u}}_{0}$) and nearby eigenvalue approximation normalized wave fields in the FFLW (*a*) and FFFW (*b*) for $\tilde{\kappa }\delta =0.5$. (Online version in colour.)

A similar investigation can be carried out for the nearby eigenvalue approximation of the field. As can be seen in figure 6, the approximation is good even for a value of $\tilde{\kappa }\delta =0.5$ that is not particularly small, and for which the agreement of the simple eigenvalue zeroth-order field is poor.

**Figure 7.**
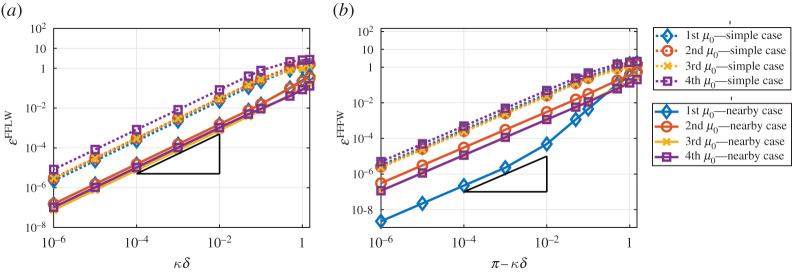
Loglog plot of the error between the exact (Bloch–Floquet) and the homogenized fields for various values of *κδ* with k=1 and m=0.5. (*a*) FFLW. (*b*) FFFW. The slope depicted by a black triangle denotes a $O(\tilde{\kappa }\delta )$. Dotted (resp. plain) lines correspond to the simple (resp. nearby) eigenvalue approximations. (Online version in colour.)

In figure 7, we plot the error between the exact field and the *homogenized* fields obtained using both the simple and nearby eigenvalue approximations. Due to the periodicity properties of $u_{\delta }$ and ${\hat{u}}_{0}$ and (6.7), the error is relatively easy to compute in the simple eigenvalue case and is defined as follows:

6.8 $$\text{(FFLW) : }E_{\text{simple}}^{\text{FFLW}}=\max_{R}|u_{\delta }(\delta y)-u_{0}(\delta y,y)|=\max_{\left(0,1\right)}|u_{\delta }(\delta y)-{\hat{u}}_{0}(y)|$$

and

6.9 $$\text{(FFFW) : }E_{\text{simple}}^{\text{FFFW}}=\max_{R}|u_{\delta }(\delta y)-u_{0}(\delta y,y)|=\max_{\left(0,1\right)}|u_{\delta }(\delta y)-{\text{e}}^{-\text{i}\pi y}{\hat{u}}_{0}(y)|.$$

In the nearby eigenvalue approximation, using (4.1), (5.7) and (4.13), the errors can be written as

6.10 $$\text{(FFLW) : }E_{\text{nearby}}^{\text{FFLW}}=\max_{\left(0,1\right)}\left|u_{\delta }(\delta y)-\left(\frac{-\langle \beta w_{0}\rangle }{\left(mB_{1}^{2}+C_{1}\right)}\frac{i\tilde{\kappa }\delta }{\delta \mu_{1}^{2}}{\hat{u}}_{0}^{\left(1\right)}(y)+{\hat{u}}_{0}^{\left(2\right)}(y)\right)\right|$$

and

6.11 $$\text{(FFFW) : }E_{\text{nearby}}^{\text{FFFW}}=\max_{\left(0,1\right)}\left|u_{\delta }(\delta y)-{\text{e}}^{-\text{i}\pi y}\left(\frac{-\langle \beta w_{0}\rangle }{\left(mB_{1}^{2}+C_{1}\right)}\frac{i\tilde{\kappa }\delta }{\delta \mu_{1}^{2}}{\hat{u}}_{0}^{\left(1\right)}(y)+{\hat{u}}_{0}^{\left(2\right)}(y)\right)\right|.$$

One can see that we recover the expected behaviour $u_{\delta }(\delta y)=u_{0}(\delta y,y)+O(\tilde{\kappa }\delta )$, but that the nearby eigenvalue approximation performs much better for the whole range of values of $\tilde{\kappa }\delta$ used in figure 7.

**Figure 8.**
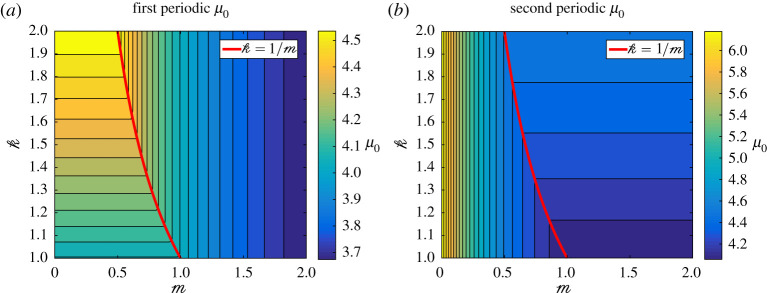
Filled contour plot of the first (*a*) and second (*b*) periodic eigenvalues *μ*_0_ as m and k vary. The thick red line represents the locus of double eigenvalues, while the thin black lines are isolines of *μ*_0_. (Online version in colour.)

We now endeavour to study how the eigenvalues *μ*_0_ depend on m and k. In order to visualize this we display a heat map of the first and second FFLW (periodic) $\mu_{0}(m,k)$ in figure 8. One can clearly see two distinct regions, on the left and on the right of the curve k=1/m. On each side of these curves, the eigenvalues depend solely on one of the two parameters (m,k), which correspond to either the top or the bottom line of $M^{\mathrm{mo}}$ being zero. On the curve k=1/m, both lines of $M^{\mathrm{mo}}$ are zero, and hence, the eigenvalue *μ*_0_ is a double eigenvalue.

**Figure 9.**
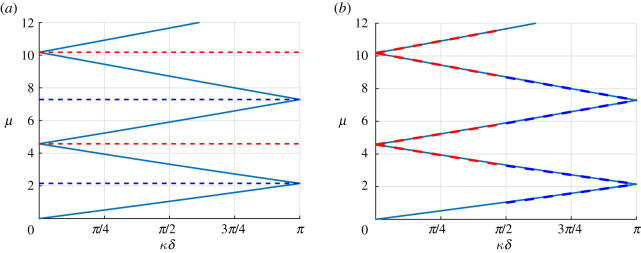
Dispersion diagram for the monolayer with k=2 and m=0.5 corresponding to double eigenvalues. (*a*) The red (resp. blue) dashed lines are the periodic (resp. antiperiodic) eigenvalues *μ*_0_ calculated by finding the roots of (6.4). (*b*) The red (resp. blue) dashed lines are the periodic (resp. antiperiodic) resulting first-order approximations (4.15). (Online version in colour.)

The other eigenvalues in the the FFLW (periodic) and FFFW (antiperiodic) cases have very similar heatmaps, in particular they are all double eigenvalues when k=1/m, as displayed on the dispersion diagram in figure 9*a*.

In the case of a double eigenvalue, we have to follow the procedure of §4 by representing *u*_0_ as in (4.1). We hence need to find two independent solutions ${\hat{u}}_{0}^{\left(1,2\right)}$ of (3.1), which can both be written ${\hat{u}}_{0}^{\left(1,2\right)}(y)=A^{\left(1,2\right)}\cos(\mu_{0}y)+B^{\left(1,2\right)}\sin(\mu_{0}y)$. Because of the dimension of the system, any two independent vectors (*A*^(1)^, *B*^(1)^)^*T*^ and (*A*^(2)^, *B*^(2)^)^*T*^ would work, and we can hence choose (*A*^(1)^, *B*^(1)^)^*T*^ = (1, 0)^*T*^ and (*A*^(2)^, *B*^(2)^)^*T*^ = (0, 1)^*T*^. As seen in §4, the two functions $U_{0}^{\left(1,2\right)}(x)$ appearing in (4.1) satisfy the first-order ODE system (4.9), where in our case we have

$$\begin{array}{rl} B_{1} & =\langle \langle {\hat{u}}_{0}^{\left(1\right)}\rangle \rangle =\frac{1\pm \cos(\mu_{0})}{2},\;B_{2}=\langle \langle {\hat{u}}_{0}^{\left(2\right)}\rangle \rangle =\frac{\pm \sin(\mu_{0})}{2},\;C_{1}=\langle \alpha {\left({\hat{u}}_{0}^{\left(1\right)}\right)}^{2}\rangle =\frac{\mu_{0}+\sin(\mu_{0})\cos(\mu_{0})}{2\mu_{0}},\\ D & =\langle \alpha {\hat{u}}_{0}^{\left(1\right)}{\hat{u}}_{0}^{\left(2\right)}\rangle =\frac{\sin^{2}(\mu_{0})}{2\mu_{0}},\;\langle \beta w_{0}\rangle =\mu_{0},\;C_{2}=\langle \alpha {\left({\hat{u}}_{0}^{\left(2\right)}\right)}^{2}\rangle =\frac{\mu_{0}-\sin(\mu_{0})\cos(\mu_{0})}{2\mu_{0}}. \end{array}$$

Using these, one can easily compute the associated eigenvalues *λ*_1,2_ and eigenvectors $U_{\lambda_{1,2}}$ via (4.10) and (4.11). Note that in this case, one can show that

$$mB_{1}B_{2}+D=0\;\text{and}\;mB_{1}^{2}+C_{1}=mB_{2}^{2}+C_{2},$$

so that the eigenvalues *λ*_1,2_ and eigenvectors $U_{\lambda_{1,2}}$ of the matrix of the ODE system are simply

$$\lambda_{j}=i{\left(-1\right)}^{j}(mB_{1}^{2}+C_{1})\;\text{and}\;U_{\lambda_{j}}={\left(-i{\left(-1\right)}^{j},1\right)}^{T},$$

and $T_{d}=\mu_{0}/(mB_{1}^{2}+C_{1})$. We can hence superpose the resulting linear approximation (4.15) onto the dispersion diagram, revealing an excellent fit, as can be seen in figure 9*b*. It is quite remarkable that in this case, every eigenvalue *μ*_0_ corresponds to Dirac points. In fact this can be understood by considering a homogeneous material with only one spring-mass interface. Upon sending a wave onto this interface, one can naturally derive a coefficient of reflection Ref(μ) and a coefficient of transmission Trans(μ). It turns out that

$$\begin{array}{rl} \mathrm{Ref}(\mu ) & =\frac{-i\mu ((1/k)-m)}{2(1-(m\mu^{2}/4k))-i\mu ((1/k)+m)}\;\text{and}\\ \mathrm{Trans}(\mu ) & =\frac{2(1+(m\mu^{2}/4k))}{2(1-(m\mu^{2}/4k))-i\mu ((1/k)+m)}, \end{array}$$

and therefore the reflection coefficient is zero if and only if the condition k=1/m is satisfied. Hence, in the periodic medium considered, no internal reflection can be present, no destructive/constructive interference can take place and no band gaps occur.

### (b) Bilayer

We now consider the case of a bilayer material characterized by the phase fraction *r* ∈ (0, 1), and hence provide the imperfect interface extension to the example given in [6]. The unit cell is made up of two homogeneous materials. The first one has a length *rh*, density *ρ*_1_ and Young’s modulus *E*_1_, while the second has length (1 − *r*) *h*, density *ρ*_2_ and Young’s Modulus *E*_2_. The two respective wave speeds are $c_{1}=\sqrt{E_{1}/\rho_{1}}$ and $c_{2}=\sqrt{E_{2}/\rho_{2}}$. The important non-dimensional functions *α* and *β* are hence defined by

**Figure 10.**
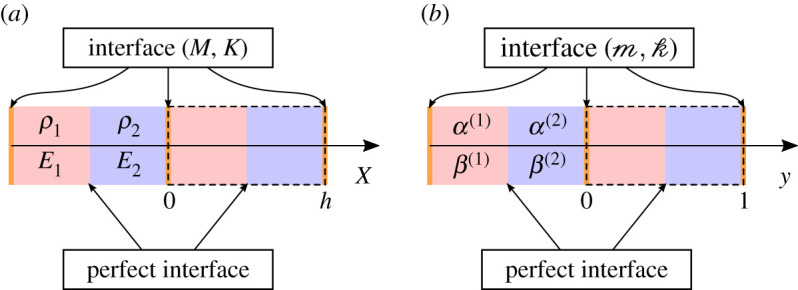
Geometry of the bilayer problem (*a*) and non-dimensional (*b*) settings. (Online version in colour.)

6.12 $$\alpha (y)=\left\{ \begin{array}{ll} \alpha^{\left(1\right)}=\frac{\rho_{1}}{\rho^{⋆}} & \text{ for }y\in (0,r),\\ \alpha^{\left(2\right)}=\frac{\rho_{2}}{\rho^{⋆}} & \text{ for }y\in (r,1), \end{array}\right.\;\text{and}\;\beta (y)=\left\{ \begin{array}{ll} \beta^{\left(1\right)}=\frac{E_{1}}{E^{⋆}} & \text{ for }y\in (0,r),\\ \beta^{\left(2\right)}=\frac{E_{2}}{E^{⋆}} & \text{ for }y\in (r,1), \end{array}\right.$$

where $\rho^{⋆}=r\rho_{1}+(1-r)\rho_{2}$ and $E^{⋆}={\left(r/E_{1}+(1-r)/E_{2}\right)}^{-1}$. The interface at *y* = *r* is assumed perfect, and the geometry of this physical problem is summarized in figure 10.

The classic Bloch–Floquet analysis will, in this case, give the following dispersion relation

**Figure 11.**
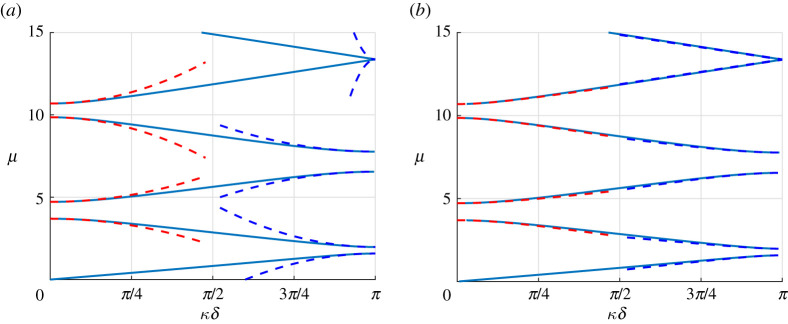
Non-dimensional dispersion diagram for the bilayer with *ρ*_1_ = 1200 kg m^−3^, *ρ*_2_ = 1800 kg m^−3^, *c*_1_ = 2800 m s^−1^, *c*_2_ = 3500 m s^−1^, $E_{1}=\rho_{1}c_{1}^{2}$, $E_{2}=\rho_{2}c_{2}^{2}$, *M* = 2 × 10^4^ kg m^2^, *K* = 2.45 × 10^9^ Pa m^−1^, *r* = 0.202 and *h* = 10 m, corresponding to k≈1.41 and m≈1.19, *α*^(1)^ ≈ 0.71, *α*^(2)^ ≈ 1.071, *β*^(1)^ ≈ 0.54 and *β*^(2)^ ≈ 1.27. The red (resp. blue) dashed lines are the periodic (resp. antiperiodic) second-order approximations (3.31) using the computed values of *T* (*a*) and nearby eigenvalue approximations (5.9) using the computed values of *T*_*d*_ and *δγ* (*b*). (Online version in colour.)

6.13 $$\begin{array}{rl} \cos(\kappa \delta ) & =\frac{1}{1+\frac{m\mu^{2}}{4k}}[\left(1-\frac{m\mu^{2}}{4k}\right)\left(C_{1}C_{2}-\frac{1}{2}\left(\frac{Z_{1}}{Z_{2}}+\frac{Z_{2}}{Z_{1}}\right)S_{1}S_{2}\right)\\ & \;\;\;-\frac{m\mu }{2}\left(\frac{S_{1}C_{2}Z^{⋆}}{Z_{1}}+\frac{S_{2}C_{1}Z^{⋆}}{Z_{2}}\right)-\frac{\mu }{2k}\left(\frac{Z_{1}S_{1}C_{2}}{Z^{⋆}}+\frac{Z_{2}S_{2}C_{1}}{Z^{⋆}}\right)], \end{array}$$

where $Z^{⋆}=\rho^{⋆}c^{⋆}$, and *C*_*i*_ = cos (*μH*_*i*_), *S*_*i*_ = sin (*μH*_*i*_), $H_{1}=rc^{⋆}/c_{1}$, $H_{2}=(1-r)c^{⋆}/c_{2}$ and, naturally, $c^{⋆}=\sqrt{E^{⋆}/\rho^{⋆}}$ . As per the monolayer case, the dispersion diagram displays band gaps as can be seen in figure 11. We will now apply the high-frequency homogenization technique to derive an analytical approximation to the higher branches of the diagram and to the associated wave fields.

Using (3.1)*a* and (3.4), we find that ${\hat{u}}_{0}$ should satisfy

6.14 $$\begin{array}{r} \left\{ \begin{array}{ll} {\hat{u}}_{0}^{\prime \prime }+{\left(\Omega^{\left(1\right)}\right)}^{2}{\hat{u}}_{0}=0 & \text{ on }(0,r),\\ {\hat{u}}_{0}^{\prime \prime }+{\left(\Omega^{\left(2\right)}\right)}^{2}{\hat{u}}_{0}=0 & \text{ on }(r,1), \end{array}\right.\text{so that}\;\left\{ \begin{array}{ll} {\hat{u}}_{0}(y)=A^{\left(1\right)}\cos(\Omega^{\left(1\right)}y)+B^{\left(1\right)}\sin(\Omega^{\left(1\right)}y) & \text{ on }(0,r),\\ {\hat{u}}_{0}(y)=A^{\left(2\right)}\cos(\Omega^{\left(2\right)}y)+B^{\left(2\right)}\sin(\Omega^{\left(2\right)}y) & \text{ on }(r,1), \end{array}\right. \end{array}$$

where *A*^(1,2)^ and *B*^(1,2)^ are some constants to be determined, and $\Omega^{\left(1,2\right)}=\mu_{0}\sqrt{\alpha^{\left(1,2\right)}/\beta^{\left(1,2\right)}}=\mu_{0}(c^{⋆}/c_{1,2})$. The interface at *y* = *r* is assumed perfect, and hence, ${\hat{u}}_{0}$ is also subject to the interface conditions

$$\left\{ \begin{array}{l} {\hat{u}}_{0}(r^{-})={\hat{u}}_{0}(r^{+}),\\ \beta^{\left(1\right)}{\hat{u}}_{0}^{\prime }(r^{-})=\beta^{\left(2\right)}{\hat{u}}_{0}^{\prime }(r^{+}), \end{array}\right.\;\text{and}\;\left\{ \begin{array}{l} {\hat{u}}_{0}(0^{+})\mp {\hat{u}}_{0}(1^{-})=\frac{1}{2k}(\beta^{\left(1\right)}{\hat{u}}_{0}^{\prime }(0^{+})\pm \beta^{\left(2\right)}{\hat{u}}_{0}^{\prime }(1^{-})),\\ \beta^{\left(1\right)}{\hat{u}}_{0}^{\prime }(0^{+})\mp \beta^{\left(2\right)}{\hat{u}}_{0}^{\prime }(1^{-})=-\frac{m\mu_{0}^{2}}{2}({\hat{u}}_{0}(0^{+})\pm {\hat{u}}_{0}(1^{-})), \end{array}\right.$$

where here and throughout this section, Notation 2.7 is being used. This results in a system of the form

6.15 $$M^{\mathrm{bi}}{\left(A^{\left(1\right)},B^{\left(1\right)},A^{\left(2\right)},B^{\left(2\right)}\right)}^{T}={\left(0,0,0,0\right)}^{T},$$

where the 4 × 4 matrix $M^{\mathrm{bi}}$ is given by

$$\begin{array}{rl} M^{\mathrm{bi}} & =(\begin{array}{cc} 1 & -\frac{\beta^{\left(1\right)}\Omega^{\left(1\right)}}{2k}\\ -\frac{m\mu_{0}^{2}}{2} & -\beta^{\left(1\right)}\Omega^{\left(1\right)}\\ -\cos(\Omega^{\left(1\right)}r) & -\sin(\Omega^{\left(1\right)}r)\\ \beta^{\left(1\right)}\Omega^{\left(1\right)}\sin(\Omega^{\left(1\right)}r) & -\beta^{\left(1\right)}\Omega^{\left(1\right)}\cos(\Omega^{\left(1\right)}r) \end{array}\\ & \;\begin{array}{cc} \mp \cos(\Omega^{\left(2\right)})\pm \frac{\beta^{\left(2\right)}\Omega^{\left(2\right)}\sin(\Omega^{\left(2\right)})}{2k} & \mp \sin(\Omega^{\left(2\right)})\mp \frac{\beta^{\left(2\right)}\Omega^{\left(2\right)}\cos(\Omega^{\left(2\right)})}{2k}\\ \mp \beta^{\left(2\right)}\Omega^{\left(2\right)}\sin(\Omega^{\left(2\right)})\mp \frac{m\mu_{0}^{2}}{2}\cos(\Omega^{\left(2\right)}) & \pm \beta^{\left(2\right)}\Omega^{\left(2\right)}\cos(\Omega^{\left(2\right)})\mp \frac{m\mu_{0}^{2}}{2}\sin(\Omega^{\left(2\right)})\\ \cos(\Omega^{\left(2\right)}r) & \sin(\Omega^{\left(2\right)}r)\\ -\beta^{\left(2\right)}\Omega^{\left(2\right)}\sin(\Omega^{\left(2\right)}r) & \beta^{\left(2\right)}\Omega^{\left(2\right)}\cos(\Omega^{\left(2\right)}r) \end{array}). \end{array}$$

The equation (6.15) can only have non-trivial solutions if $\det(M^{\mathrm{bi}})=0$, which gives a relation of the form

6.16 $$D^{\mathrm{bi}}(\mu_{0};m,k,\beta^{\left(1\right)},\beta^{\left(2\right)},\Omega^{\left(1\right)},\Omega^{\left(2\right)},r)=0,$$

the roots of which correspond to the eigenvalues *μ*_0_. The numerically computed eigenvalues coincide with the edges of the Brillouin zone on the dispersion diagram in figure 11. To obtain ${\hat{u}}_{0}$, we find a vector in $\ker(M^{\mathrm{bi}})$ using the null function in Matlab, and use it as the coefficients (*A*^(1)^, *B*^(1)^, *A*^(2)^, *B*^(2)^).

We now need to find $v_{1}$ to obtain a second-order approximation. Since it is a solution to the same equation (3.1)*a*, we can write

6.17 $$v_{1}(y)=\left\{ \begin{array}{ll} C^{\left(1\right)}\cos(\Omega^{\left(1\right)}y)+D^{\left(1\right)}\sin(\Omega^{\left(1\right)}y) & \text{on}\;(0,r),\\ C^{\left(2\right)}\cos(\Omega^{\left(2\right)}y)+D^{\left(2\right)}\sin(\Omega^{\left(2\right)}y) & \text{on}\;(r,1). \end{array}\right.$$

Using remark 3.1 for the conditions at the unit cell interfaces, and remembering that, according to remark 3.2, both $v_{1}$ and $\beta v_{1}^{\prime }$ should be continuous at *y* = *r*, one obtains a system of the form $M^{\mathrm{bi}}{\left(C^{\left(1\right)},D^{\left(1\right)},C^{\left(2\right)},D^{\left(2\right)}\right)}^{T}=b^{\mathrm{bi}}$, where

$$b^{\mathrm{bi}}={\left(\mp {\hat{u}}_{0}(1^{-})\mp \frac{1}{2k}\beta^{\left(2\right)}{\hat{u}}_{0}^{\prime }(1^{-}),\mp \frac{m\mu_{0}^{2}}{2}{\hat{u}}_{0}(1^{-})\pm \beta^{\left(2\right)}{\hat{u}}_{0}^{\prime }(1^{-}),0,0\right)}^{T}.$$

Because the matrix $M^{\mathrm{bi}}$ is singular, we compute (*C*^(1)^, *D*^(1)^, *C*^(2)^, *D*^(2)^)^*T*^ via the Moore–Penrose Pseudo-inverse [29]. Once v_1_ is found, the resulting value of *T* is directly obtained using (3.24). The resulting approximations $\mu \approx \mu_{0}+(T/2\mu_{0}){\left(\tilde{\kappa }\delta \right)}^{2}$ are superposed to the Bloch–Floquet diagram in figure 11*a*, and one can see that they approximate the branches well in the vicinity of the edges of the band gaps of the dispersion diagram. It is apparent from figure 11 that the highest antiperiodic eigenvalue displayed seems to be a double eigenvalue (in fact we will see further that it is not exactly a double eigenvalue), and that the approximation is particularly short-lived in this neighbourhood. Having computed all the eigenvalues and eigenfunctions, we can once again evaluate the nearby eigenvalue approximation (5.9). It is displayed in figure 11*b*, and as can clearly be seen, its agreement with the dispersion diagram is much longer-lived that of the simple eigenvalue approximation, even more so for the near-double eigenvalue.

**Figure 12.**
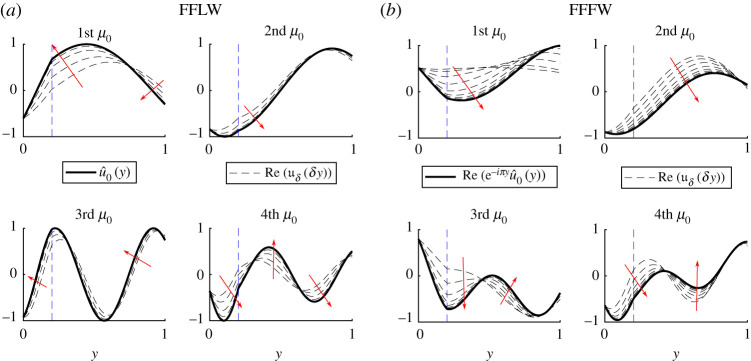
Illustration of the convergence of the method by comparing ${\hat{u}}_{0}$ and $u_{\delta }(\delta y)$ in the FFLW (*a*) and the FFFW (*b*) cases. We plotted $u_{\delta }$ for 20 values of $\tilde{\kappa }\delta$ equidistributed in the log scale between 10^−5^ and 1. The red arrows indicate how $u_{\delta }(\delta y)$ is changing as $\tilde{\kappa }\delta \to 0$, while the vertical dashed blue line indicates the position of the perfect interface between the two homogeneous materials. (Online version in colour.)

Using the Bloch–Floquet analysis, in a very similar way to the monolayer case, we can have access to the exact standing wave field *u*_*δ*_ (*x*) = *u*_*δ*_ (*δy*), and we normalize it such that *u*_*δ*_ (0^+^) = *u*_0_ (0, 0^+^). As per the monolayer case, and because of (6.7), we illustrate the convergence of the simple eigenvalue method by comparing $u_{\delta }(\delta y)$ and ${\hat{u}}_{0}(y)$ for various values of *κδ* in figure 12.

**Figure 13.**
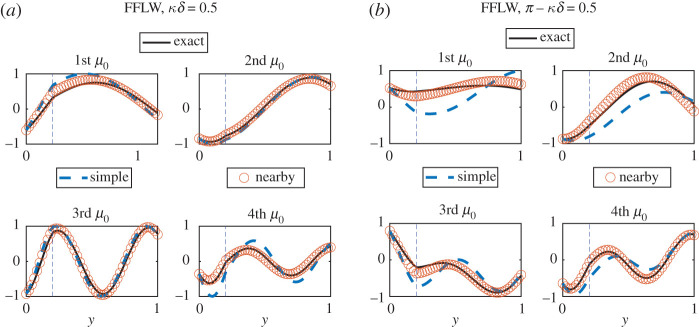
Superposition of real parts of the exact ($u_{\delta }$), simple eigenvalue zeroth-order (${\hat{u}}_{0}$) and nearby eigenvalue approximation normalized wave fields in the FFLW (*a*) and FFFW (*b*) for $\tilde{\kappa }\delta =0.5$ and for the same parameters used in figure 11. (Online version in colour.)

A similar investigation can be carried out for the nearby eigenvalue approximation. As can be seen in figure 13, the approximation is good even for a value of $\tilde{\kappa }\delta =0.5$ that is not particularly small, and for which the agreement of the simple eigenvalue zeroth-order field is poor.

**Figure 14.**
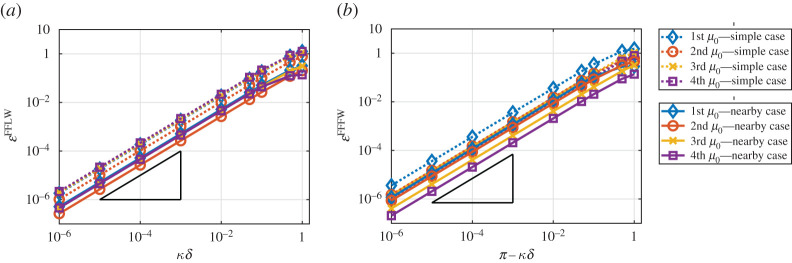
Loglog plot of the error between the exact (Bloch–Floquet) and the homogenized fields for various values of *κδ* and for the same parameters used in figure 11. (*a*) FFLW. (*b*) FFFW. The slope depicted by a black triangle denotes a $O(\tilde{\kappa }\delta )$. Dotted (resp. plain) lines correspond to the simple (resp. nearby) eigenvalue approximation. (Online version in colour.)

In figure 14, we plot the error between the exact field and the *homogenized* fields obtained using both the simple and nearby eigenvalue approximations. The errors can be expressed as in (6.8)–(6.11). Again, one can see that we recover the expected behaviour $u_{\delta }(\delta y)=u_{0}(\delta y,y)+O(\tilde{\kappa }\delta )$ , but that the nearby eigenvalue approximation performs better for the whole range of values of $\tilde{\kappa }\delta$ used in figure 14.

As for the monolayer example displayed in figure 9, it is possible to find physical parameters such that all the eigenvalues become simultaneously double eigenvalues (Dirac points). In order to do so one needs to ensure that k=1/m, and that *ρ*_1_ *c*_1_ = *ρ*_2_ *c*_2_. The first condition imposes that the reflection coefficient due to the imperfect interface is zero, and the second imposes that the two homogeneous materials are “impedance matched” so that no reflection occurs from their perfect interface either.

**Figure 15.**
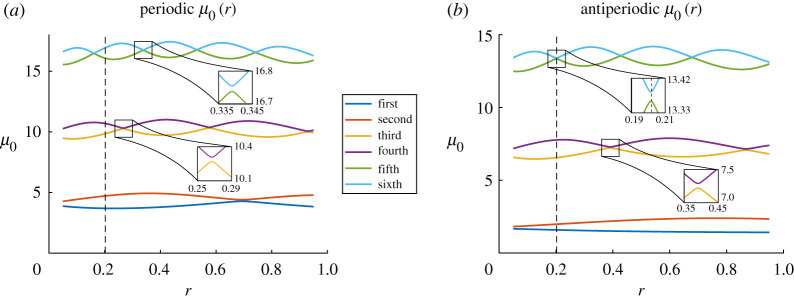
Evolution of the first six FFLW (*a*) and FFFW (*b*) eigenvalues *μ*_0_ for the exact same parameters as those used in figure 11, but for *r* ∈ (0.05, 0.95). A vertical dashed line represents the value of *r* used in figure 11. Zoom boxes are provided to show that near the almost-Dirac points, the eigenvalues remain simple. (Online version in colour.)

However, for the bilayer, it appears that certain parameters lead to only two of the eigenvalues merging into a double eigenvalue, as appears to be the case in figure 11. In order to visualize this phenomena, one could look at the evolution of the eigenvalues *μ*_0_ for fixed physical parameters, but for varying *r* within (0, 1). The results are displayed in figure 15, and it seems that double eigenvalues or near-double eigenvalues may occur for some specific values of *r*, though, in this case, all the eigenvalues do not become double simultaneously. In fact, as illustrated in figure 15, if one zooms in on the areas of the graphs where eigenvalues seem to coincide, it appears that the curves do not actually touch each other. We will call these points *almost-Dirac points*. As highlighted above, the nearby eigenvalue approximation to the dispersion diagram is excellent for such almost-Dirac points, while the simple eigenvalue method leads to a very short-lived approximation, see figure 11.

**Figure 16.**
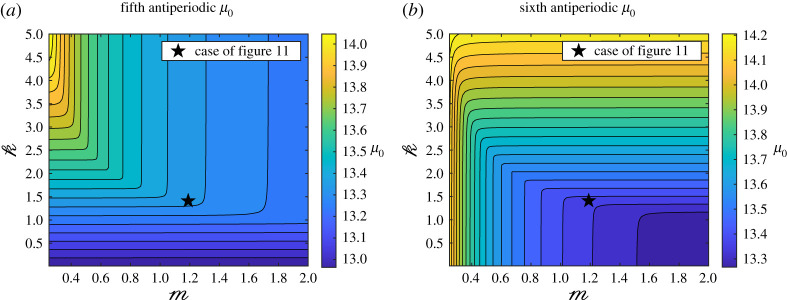
Filled contour plot of the variations of the fifth (*a*) and sixth (*b*) antiperiodic eigenvalues in the bilayer case for the same values of *r*, *α*^(1,2)^ and *β*^(1,2)^ used in figure 11. The black star corresponds to the values of m and k used in figure 11. (Online version in colour.)

To explore the parameter space further, we will keep the values of *r*, *α*^(1,2)^ and *β*^(1,2)^ used in figure 11 and study the variation of the fifth and sixth antiperiodic eigenvalues *μ*_0_ that correspond to an almost-Dirac point according to figure 15. As can be seen in figure 16, in this case, we observe a similar behaviour as that of figure 8, where two distinct regions seem to be separated by a smooth curve, on which the values of m and k chosen in figure 11 (represented by a black star) seem to lie.

All in all, it seems to be the case, that for a given integer *j*, the *j*th eigenvalue is double on some manifold given by $F_{j}(\rho_{1},\rho_{2},E_{1},E_{2},M,K,r)=0$ for some function $F_{j}$, though finding an analytical expression for $F_{j}$ is beyond the scope of the present work.

## 7. Conclusion

In this work, we have extended the technique of high-frequency homogenization to one-dimensional periodic media with linear imperfect interfaces of the spring-mass type. The extension was not direct, and many of the proofs for the classic case needed to be extended in order to deal with the extra technical difficulties arising from an imperfect interface. We have also described how the technique should be modified in the specific case of Dirac points within the dispersion diagram, and also proposed a uniform approximation taking into consideration competing nearby eigenvalues.

We have illustrated the validity of our theoretical development with the two examples of monolayered and bilayered materials. In the case of the monolayered material, we quantified the error between the exact and the homogenized fields, and we found a simple condition on the non-dimensional stiffness and mass values k and m for all the points at the edges of the Brillouin zone to become Dirac points. Similar conditions were given in the bilayered case. Moreover, in the bilayered case, almost-Dirac points have been identified while exploring the parameter space (much larger than that of the monolayered material). For both examples considered, we found that the nearby eigenvalue approximation led to a much longer lived approximation to both the dispersion diagram and the wave fields. This formulation is also convenient because it does not break down when two eigenvalues merge, unlike the simple eigenvalue approximation. As mentioned previously, one of the advantages of high-frequency homogenization is that it works even when the dispersion diagrams cannot be obtained analytically or are computationally intricate to obtain. In such cases, this high-frequency homogenization approach may, for example, provide a way of back-engineering the values of m and k describing imperfect interface, or pick specific material properties and contacts in order to ensure the presence of Dirac points, which are known to display very interesting physical properties.

In the future, we hope to be able to push the asymptotics presented in this paper to higher order, i.e. to propose first- and maybe second-order corrections to the leading-order wave fields exhibited in the present work.

## Data accessibility

This article has no additional data.

## Appendix A. On the self-adjointness of (3.1)

The system (3.1) is the eigenvalue problem that can be formulated as follows. Find *λ* such that L[f]=λf with ⟦f⟧=(1/k)⟨⟨β(df/dy)⟩⟩ (1st jump) and ⟦β(df/dy)⟧=−mλ⟨⟨f⟩⟩ (2nd jump), for some function *f* that is periodic (FFLW) or antiperiodic (FFFW). It is somewhat inconvenient for *λ* to appear in the second jump, so we rewrite it as ⟦β(df/dy)⟧=−m⟨⟨L[f]⟩⟩ (2nd jump). We will now show that the operator L, together with the jump and periodicity conditions, is both symmetric and non-negative for the inner product (3.2), that is ⟨L[f],g⟩=⟨f,L[g]⟩ and ⟨L[f],f⟩≥0 for any functions *f* and *g* satisfying both jump conditions and both periodic (FFLW) or antiperiodic (FFFW). To prove symmetry, we will show that the quantity Sym(f,g)=⟨L[f],g⟩−⟨f,L[g]⟩ is zero. First by definition of the inner product (3.2) we have

A 1 $$\begin{array}{rl} \mathrm{Sym}(f,g) & \overset{\text{2nd jump}}{=}\langle \alpha L[f]\bar{g}\rangle +m\langle \langle L[f]\rangle \rangle \langle \langle \bar{g}\rangle \rangle -\langle \alpha f\bar{L[g]}\rangle -m\langle \langle f\rangle \rangle \langle \langle \bar{L[g]}\rangle \rangle \\ & \overset{\text{2nd jump}}{\underset{\text{def of}L}{=}}\left\langle f\frac{\text{d}}{\text{d}y}\left(\beta \frac{\text{d}\bar{g}}{\text{d}y}\right)-\frac{\text{d}}{\text{d}y}\left(\beta \frac{\text{d}f}{\text{d}y}\right)\bar{g}\right\rangle +\langle \langle f\rangle \rangle \left[\left[\beta \frac{\text{d}\bar{g}}{\text{d}y}\right]\right]-\left[\left[\beta \frac{\text{d}f}{\text{d}y}\right]\right]\langle \langle \bar{g}\rangle \rangle . \end{array}$$

Now, using the fact that $f(\text{d}/\text{d}y)(\beta (\text{d}\bar{g}/\text{d}y))-(\text{d}/\text{d}y)(\beta (\text{d}f/\text{d}y))\bar{g}=(\text{d}/\text{d}y)(f\beta (\text{d}\bar{g}/\text{d}y)-\beta (\text{d}f/\text{d}y)\bar{g})$, (1) becomes

$$\begin{array}{rl} \mathrm{Sym}(f,g) & \underset{\text{Lemma 2.6}}{=}\left\langle \frac{\text{d}}{\text{d}y}\left(f\beta \frac{\text{d}\bar{g}}{\text{d}y}-\beta \frac{\text{d}f}{\text{d}y}\bar{g}\right)\right\rangle +\langle \langle f\rangle \rangle \left[\left[\beta \frac{\text{d}\bar{g}}{\text{d}y}\right]\right]-\left[\left[\beta \frac{\text{d}f}{\text{d}y}\right]\right]\langle \langle \bar{g}\rangle \rangle \\ & \underset{\text{Lemma 2.5}}{=}-\left[\left[f\beta \frac{\text{d}\bar{g}}{\text{d}y}-\beta \frac{\text{d}f}{\text{d}y}\bar{g}\right]\right]+\langle \langle f\rangle \rangle \left[\left[\beta \frac{\text{d}\bar{g}}{\text{d}y}\right]\right]-\left[\left[\beta \frac{\text{d}f}{\text{d}y}\right]\right]\langle \langle \bar{g}\rangle \rangle \\ & \underset{\text{Lemma 2.5}}{=}\left\langle \left\langle \beta \frac{\text{d}f}{\text{d}y}\right\rangle \right\rangle \text{⟦}\bar{g}\text{⟧}-\text{⟦}f\text{⟧}\left\langle \left\langle \beta \frac{\text{d}\bar{g}}{\text{d}y}\right\rangle \right\rangle \underset{\text{1st jump}}{=}k\text{⟦}f\text{⟧⟦}\bar{g}\text{⟧}-k\text{⟦}f\text{⟧⟦}\bar{g}\text{⟧}=0, \end{array}$$

and hence the problem is symmetric. Now, by definition of the inner product (3.2), we have

A 2 $$\langle L[f],f\rangle =\langle \alpha L[f]\bar{f}\rangle +m\langle \langle L[f]\rangle \rangle \langle \langle \bar{f}\rangle \rangle \overset{\text{2nd jump}}{\underset{\text{def of}L}{=}}-\left\langle \frac{\text{d}}{\text{d}y}\left(\beta \frac{\text{d}f}{\text{d}y}\right)\bar{f}\right\rangle -\left[\left[\beta \frac{\text{d}f}{\text{d}y}\right]\right]\langle \langle \bar{f}\rangle \rangle .$$

Now, using the fact that $(\text{d}/\text{d}y)(\beta (\text{d}f/\text{d}y))\bar{f}=(\text{d}/\text{d}y)(\beta (\text{d}f/\text{d}y)\bar{f})-\beta |\text{d}f/\text{d}y{|}^{2}$, (2) becomes

$$\begin{array}{rl} \left\langle L[f],f\right\rangle & \underset{\text{Lemma 2.6}}{=}\left\langle \beta {\left|\frac{\text{d}f}{\text{d}y}\right|}^{2}\right\rangle -\left\langle \frac{\text{d}}{\text{d}y}\left(\beta \frac{\text{d}f}{\text{d}y}\bar{f}\right)\right\rangle -\left[\left[\beta \frac{\text{d}f}{\text{d}y}\right]\right]\langle \langle \bar{f}\rangle \rangle \\ & \underset{\text{Lemma 2.6}}{=}\left\langle \beta {\left|\frac{\text{d}f}{\text{d}y}\right|}^{2}\right\rangle +\left[\left[\beta \frac{\text{d}f}{\text{d}y}\bar{f}\right]\right]-\left[\left[\beta \frac{\text{d}f}{\text{d}y}\right]\right]\langle \langle \bar{f}\rangle \rangle \\ & \underset{\text{Lemma 2.5}}{=}\left\langle \beta {\left|\frac{\text{d}f}{\text{d}y}\right|}^{2}\right\rangle +\left\langle \left\langle \beta \frac{\text{d}f}{\text{d}y}\right\rangle \right\rangle \text{⟦}\bar{f}\text{⟧}\underset{\text{1st jump}}{=}\left\langle \beta {\left|\frac{\text{d}f}{\text{d}y}\right|}^{2}\right\rangle +k|\text{⟦}f\text{⟧}{|}^{2}\geq 0, \end{array}$$

hence the problem is non-negative. Therefore, in conclusion, the operator L, together with the jump and periodicity conditions, is self-adjoint and non-negative.

## Appendix B. Bloch–Floquet analysis of the monolayer case

In this case, *u*_*δ*_ satisfies (2.5) with *α* ≡ *β* ≡ 1, hence, on (0, *δ*), we have

B 1 $$u_{\delta }(x)=A_{\mathrm{BF}}\cos\left(\frac{\mu x}{\delta }\right)+B_{\mathrm{BF}}\sin\left(\frac{\mu x}{\delta }\right)$$

and

B 2 $$u_{\delta }^{\prime }(x)=-\frac{A_{\mathrm{BF}}\mu }{\delta }\sin\left(\frac{\mu x}{\delta }\right)+\frac{B_{\mathrm{BF}}\mu }{\delta }\cos\left(\frac{\mu x}{\delta }\right),$$

subject to the two jump conditions $\text{⟦}u_{\delta }{\text{⟧}}_{0}=(\delta /k){\left\langle \langle u_{\delta }^{\prime }\rangle \right\rangle }_{0}$ and $\delta \text{⟦}u_{\delta }^{\prime }{\text{⟧}}_{0}=-m\mu^{2}{\left\langle \langle u_{\delta }\rangle \right\rangle }_{0}$, relating the value of *u*_*δ*_ and *u*_*δ*_′ at 0^+^ and 0^−^. Moreover, according to Bloch–Floquet theory, we know that we can write $u_{\delta }(x)=u_{\delta }(x)\;{\text{e}}^{\text{i}\kappa x}$, for a *δ*-periodic function $u_{\delta }$, implying that, in particular, we have *u*_*δ*_ (*δ*^−^) = *u*_*δ*_ (0^−^) e^i*κδ*^ and *u*_*δ*_′ (*δ*^−^) = *u*_*δ*_′ (0^−^) e^i*κδ*^. This, combined with the jump conditions, relates the values of *u*_*δ*_ and *u*_*δ*_′ at 0^+^ and *δ*^−^, and hence it gives two equations on $A_{\mathrm{BF}}$ and $B_{\mathrm{BF}}$. These equations can be summarized by a matrix equation of the form $M_{\mathrm{BF}}^{\mathrm{mo}}(\mu ,\kappa \delta ,m,k){\left(A_{\mathrm{BF}},B_{\mathrm{BF}}\right)}^{T}={\left(0,0\right)}^{T}$, where

$$M_{\mathrm{BF}}^{\mathrm{mo}}=\left(\begin{array}{cc} 1-{\text{e}}^{-\text{i}\kappa \delta }\cos(\mu )+\frac{\mu }{2k}\sin(\mu )\;{\text{e}}^{-\text{i}\kappa \delta } & -\sin(\mu )\;{\text{e}}^{-\text{i}\kappa \delta }-\frac{\mu }{2k}(1+\cos(\mu )\;{\text{e}}^{-\text{i}\kappa \delta })\\ -\mu \sin(\mu )\;{\text{e}}^{-\text{i}\kappa \delta }-\frac{m\mu^{2}}{2}(1+\cos(\mu )\;{\text{e}}^{-\text{i}\kappa \delta }) & -\mu (1-\cos(\mu )\;{\text{e}}^{-\text{i}\kappa \delta })-\frac{m\mu^{2}}{2}\sin(\mu )\;{\text{e}}^{-\text{i}\kappa \delta } \end{array}\right).$$

One notes that, as expected, for *κδ* = 0 or *κδ* = *π* we have $M_{\mathrm{BF}}^{\mathrm{mo}}=M^{\mathrm{mo}}$ with the relevant sign. Of course, this system has only non-trivial solutions if $\det(M_{\mathrm{BF}}^{\mathrm{mo}})=0$. A little bit of algebra shows that

$$\det(M_{\mathrm{BF}}^{\mathrm{mo}})=\mu \left(-B+2\;{\text{e}}^{-\text{i}\kappa \delta }\left(C\cos(\mu )-\frac{1}{2}\left(\frac{1}{k}+m\right)\mu \sin(\mu )\right)-B\;{\text{e}}^{-2\text{i}\kappa \delta }\right),$$

where $B=1+(m\mu^{2}/4k)$ and $C=1-(m\mu^{2}/4k)$. Equating it to zero and multiplying by ${\text{e}}^{\text{i}\kappa \delta }/(B\mu )$, this leads to the dispersion relation (6.1). For a value of *μ* satisfying the dispersion relation, we have infinitely many possible ${\left(A_{\mathrm{BF}},B_{\mathrm{BF}}\right)}^{T}$. To find one, just fix $A_{\mathrm{BF}}=1$, and use either the first or second line of $M_{\mathrm{BF}}^{\mathrm{mo}}$ to get $B_{\mathrm{BF}}$ as follows:

$$B_{\mathrm{BF}}=\frac{1+{\text{e}}^{-\text{i}\kappa \delta }(-\cos(\mu )+(\mu /2k)\sin(\mu ))}{\left(\mu /2k)+{\text{e}}^{-\text{i}\kappa \delta }(\sin(\mu )+(\mu /2k)\cos(\mu )\right)}\;\text{or}\;B_{\mathrm{BF}}=\frac{m\mu /2+{\text{e}}^{-\text{i}\kappa \delta }(\sin(\mu )+(m\mu /2)\cos(\mu ))}{-1+{\text{e}}^{-\text{i}\kappa \delta }(\cos(\mu )-(m\mu /2)\sin(\mu ))},$$

depending on which one has a non-zero denominator. It then leads to the exact Bloch–Floquet solution *u*_*δ*_ (*x*) on (0, *δ*). From this we recover $u_{\delta }(x)=u_{\delta }(x)\;{\text{e}}^{-\text{i}\kappa x}$ on (0, *δ*), which we can extend to x∈R by periodicity. We can therefore get *u*_*δ*_ (*x*) everywhere by $u_{\delta }(x)=u_{\delta }(x)\;{\text{e}}^{\text{i}\kappa x}$.
